# Single-cell multi-omics of human preimplantation embryos shows susceptibility to glucocorticoids

**DOI:** 10.1101/gr.276665.122

**Published:** 2022-09

**Authors:** Cheng Zhao, Savana Biondic, Katherine Vandal, Åsa K. Björklund, Michael Hagemann-Jensen, Theresa Maria Sommer, Jesica Canizo, Stephen Clark, Pascal Raymond, Daniel R. Zenklusen, Nicolas Rivron, Wolf Reik, Sophie Petropoulos

**Affiliations:** 1 Department of Clinical Science, Intervention and Technology, Division of Obstetrics and Gynecology, Karolinska Institutet, 14186 Stockholm, Sweden;; 2 Centre de Recherche du Centre Hospitalier de l'Université de Montréal, Axe Immunopathologie, H2X 0A9 Montréal, Canada;; 3 Département de Médecine, Université de Montréal, H3T 1J4 Montréal, Canada;; 4 Department of Cell and Molecular Biology, National Bioinformatics Infrastructure Sweden, Science for Life Laboratory, Uppsala University, SE-752 37 Uppsala, Sweden;; 5 Department of Cell and Molecular Biology, Karolinska Institutet, 171 77 Stockholm, Sweden;; 6 Institute of Molecular Biotechnology of the Austrian Academy of Sciences (IMBA), Vienna BioCenter (VBC), 1030 Vienna, Austria;; 7 Epigenetics Programme, Babraham Institute, Cambridge CB22 3AT, United Kingdom;; 8 Département de Biochimie et Médecine Moléculaire, Université de Montréal, H3T 1J4 Montréal, Canada;; 9 Wellcome Sanger Institute, Cambridge CB10 1RQ, United Kingdom;; 10 Center for Trophoblast Research, University of Cambridge, Cambridge CB2 3EG, United Kingdom

## Abstract

The preconceptual, intrauterine, and early life environments can have a profound and long-lasting impact on the developmental trajectories and health outcomes of the offspring. Given the relatively low success rates of assisted reproductive technologies (ART; ∼25%), additives and adjuvants, such as glucocorticoids, are used to improve the success rate. Considering the dynamic developmental events that occur during this window, these exposures may alter blastocyst formation at a molecular level, and as such, affect not only the viability of the embryo and the ability of the blastocyst to implant, but also the developmental trajectory of the first three cell lineages, ultimately influencing the physiology of the embryo. In this study, we present a comprehensive single-cell transcriptome, methylome, and small RNA atlas in the day 7 human embryo. We show that, despite no change in morphology and developmental features, preimplantation glucocorticoid exposure reprograms the molecular profile of the trophectoderm (TE) lineage, and these changes are associated with an altered metabolic and inflammatory response. Our data also suggest that glucocorticoids can precociously mature the TE sublineages, supported by the presence of extravillous trophoblast markers in the polar sublineage and presence of X Chromosome dosage compensation. Further, we have elucidated that epigenetic regulation—DNA methylation and microRNAs (miRNAs)—likely underlies the transcriptional changes observed. This study suggests that exposures to exogenous compounds during preimplantation may unintentionally reprogram the human embryo, possibly leading to suboptimal development and longer-term health outcomes.

It is well accepted in the field of developmental origins of health and disease (DOHaD) that the preconceptual, preimplantation, intrauterine, and early life environments can have a profound and lasting impact on the developmental trajectories and longer-term health outcomes of the offspring (Barker et al. 2006; Sinclair et al. 2007; Feuer and Rinaudo 2012; Fleming et al. 2018). The preimplantation period is considered one of the most sensitive windows in development. During this time, the zygote undergoes a series of cell divisions and differentiation events to acquire distinct gene expression profiles and three cellular fates (Blakeley et al. 2015; Petropoulos et al. 2016; Meistermann et al. 2021). These three cell types eventually give rise to the placenta and the embryo proper. As such, “insults” during this window of development may alter blastocyst development, the ability of the embryo to implant, proper placental development, and embryo formation. Indeed, animal studies examining the consequences of “insults” during this window have shown that there are both short- and long-term pathologies associated with these exposures, which include altered growth (intrauterine growth restriction, small for gestational age), abnormal organ development, and development of disease (such as obesity, diabetes, hypertension, cancer) and disorders (attention-deficit hyperactivity disorder, imprinted disorders, neurocognitive, lower IQ) (Barker et al. 2006; Watkins et al. 2008, 2010; Fleming et al. 2018).

Glucocorticoids are necessary to ensure optimal fetal development; however, in excess they result in suboptimal long-term offspring development. One source of excess preimplantation glucocorticoid exposure is maternal stress. Indeed, women undergoing infertility treatment have been reported to experience an increased level of stress and anxiety, which negatively impacts reproduction (Kee et al. 2000; Neggers et al. 2006; Cesta et al. 2018). Animal models of maternal stress have shown that preimplantation glucocorticoid exposure results in long-term developmental consequences reaching into postnatal life and adulthood, including a significant reduction in implantation sites in utero, altered behavior, lower body weight, altered metabolic function, increased fat deposits, altered HPA-axis function, suboptimal cardiovascular health, and a proinflammatory immune response (Watkins et al. 2008; Burkuš et al. 2013; Bronson and Bale 2014, 2016; Zhao et al. 2015, 2021a; Jašarević et al. 2017; Chan et al. 2018; Zhai et al. 2020). In vitro models studying the direct effects of glucocorticoid exposure on the preimplantation embryo (nonhuman) remain controversial in terms of the effects observed, but those using higher doses of glucocorticoid have reported cellular apoptosis and reduced hatching rates (Van Merris et al. 2007; González et al. 2010; Santana et al. 2014; Čikoš et al. 2019). Together these studies support the hypothesis that excess glucocorticoid exposure during the preimplantation period can have both short- and long-term adverse effects on embryo development and health outcomes.

Subfertility affects one in six couples, and despite advances in in vitro fertilization (IVF), the current rate of success remains at ∼25% (Gunby et al. 2010). As such, adjuvants, such as hormonal treatments, and additives are administered with the hope to improve the implantation and live birth rate. Synthetic glucocorticoid administration is an adjuvant therapy used to circumvent recurrent implantation failure. In one meta-analysis examining the effects of glucocorticoid treatment during ART in 1879 couples, no conclusive evidence was found supporting the use of exogenous glucocorticoids to establish pregnancy (Boomsma et al. 2012). Despite the lack of evidence supporting the effectiveness of this treatment, glucocorticoid administration remains in use and can occur during stimulation, before or after transfer, and can remain until 7 wk of gestation (Boomsma et al. 2012; Muller et al. 2016; Kaye et al. 2017). Epidemiological studies examining the longer-term outcomes of children derived following the use of preimplantation glucocorticoid therapy remain to be conducted, and its safety has not yet been completely clarified (Solano and Arck 2020). Although the majority of children derived from ART appear to be healthy, the oldest person born is around 43, making it difficult to gauge how the development and long-term health of the offspring are impacted. Further, crude assessments of the safety of additives and adjuvants are currently performed in clinic, often by morphokinetic analysis of the preimplantation embryo and if the fetus appears “normal” at birth, lacking a detailed investigation of molecular perturbations occurring in the embryo or the possibility of longer-term health consequences. Moreover, practices used during the process of ART are rapidly changing, making specific adjuvants and additives moving targets, thus studying a cohort of children retrospectively may no longer be relevant to clinical practice today. As such, prospective analysis of embryo cell health and a quantitative measure to assess the impact of particular additives and adjuvants are desperately needed to ensure that we are not unintentionally programming our future generations for the development of disease and disorders.

To our knowledge, this is the first study to comprehensively examine molecular changes in the human embryo resulting from a preimplantation “insult.” We now hypothesize that the human preimplantation embryo is susceptible to reprogramming and that excess glucocorticoid exposure will result in dysregulation of metabolic pathways. We examined the direct effect of glucocorticoid exposure, low-dose dexamethasone (DEX), from embryonic day (E) 4 (before lineage specification) until E7 (blastocyst stage after mural–polar specification has initiated). On E7, we used a single-cell multi-omics approach and integrated parallel single-cell RNA sequencing and bisulfite sequencing to explore the impact of glucocorticoid exposure on the transcriptome and methylation and used Small-seq to quantify changes on small noncoding RNAs.

## Results

### Single-cell RNA-seq transcriptome profiling and lineage classification of human preimplantation embryos

**Figure 1.**
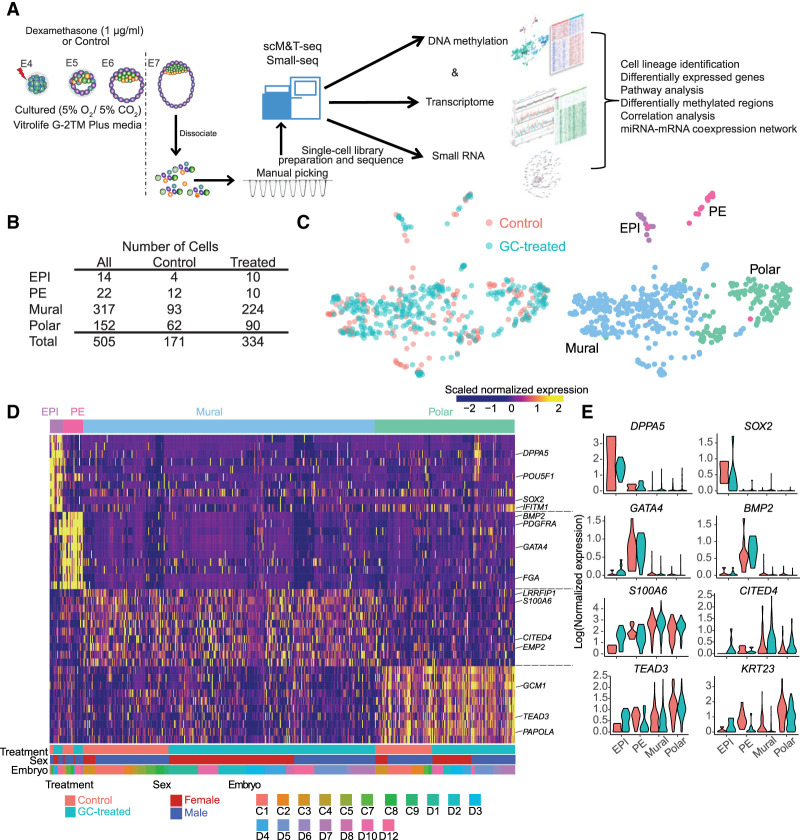
Single-cell analysis reveals cell lineages in control and glucocorticoid-exposed embryos. (*A*) Process flow diagram of single-cell sequencing data analysis on preimplantation embryos. Single cells were collected from in vitro cultured embryos on E7 (glucocorticoid-treated and control). Libraries were generated using scM&T-seq for the simultaneous measure of transcriptome and methylation and using Small-seq to measure small RNA biotypes. (*B*) The number of cells retained after quality control for scRNA-seq data. (*C*) UMAP showing individual cells from all control and glucocorticoid-exposed embryos. Cells are colored by lineages (*left*) and treatments (*right*), respectively. (*D*) Heatmap depicting the expression pattern of the top 10 marker genes (ranked by the “power” values from “roc” test) for each lineage. Names of the known marker genes are listed on the *right* side. Each row represents individual marker genes; the column represents each cell. The lineage, embryo treatment, embryo sex, and embryo identity are indicated by *upper* and *lower* panel annotation, respectively. Color in the heatmap is for the scaled expression data. (*E*) Violin plots show the expression level distributions of selected marker genes with colors indicating treatment. Lineage marker genes for each cell type are listed in Supplemental Table S2.

First, we confirmed that glucocorticoid treatment did not impact blastocyst rates, total cell number, number of cells allocated to each lineage, and overall embryo quality (Supplemental Fig. S1A,B), similar to previous reports in the mouse in which embryos were cultured in the presence of DEX (González et al. 2010). To obtain a comprehensive overview of the impact of glucocorticoid on mRNA content and gene expression, we sequenced the transcriptomes of individual blastomeres isolated from E7 embryos from both control and glucocorticoid-exposed groups. After quality control (see Supplemental Methods), we retained 505 (171 from control, 334 from treated) high-quality single-cell transcriptomes from 18 embryos, with an average of 26,051 expressed genes (reads per kilobase of transcript, per million mapped reads [RPKM] ≥ 1; average Spearman's ρ = 0.73) (Fig. 1A,B; Supplemental Fig. S1C,D; Supplemental Table S1). We then determined the sex of each embryo using X- and Y-linked gene expression as previously performed (Methods) (Supplemental Fig. S1E,F; Petropoulos et al. 2016).

To assign cell type identity, we performed data integration with E7 embryo cells from our previously published single-cell RNA-seq (scRNA-seq) data (Supplemental Fig. S2A; Petropoulos et al. 2016). Using the most variable genes across all cells as anchor genes and uniform manifold approximation and projection (UMAP), we identified the lineage populations present at E7 and carefully assigned cellular identity to the blastomeres collected; epiblast (EPI), primitive endoderm (PE), mural, and polar (Fig. 1C). We found that regardless of the dimensionality reduction technique used, cells were clearly grouped into four populations representing the EPI, PE, mural, and polar cell fates (Supplemental Fig. S2B). Further, cell identities were not biased by sex, predicted cell cycles, or embryo identity (Supplemental Fig. S2C). Due to data integration, glucocorticoid-treated and control embryos were found to overlap in the UMAP, as expected. To obtain gene signatures unique to each lineage, we then performed pair-wise differential expression analysis among the four populations (Fig. 1D; Supplemental Table S2). We verified the lineage identification by observing the expression of known candidate markers in the human embryo *DPPA5* and *SOX2* for EPI, *GATA4* and *BMP2* for PE, *S100A6* and *CITED4* for mural, and *TEAD3* and *KRT23* for polar (Fig. 1D,E; Yan et al. 2013; Blakeley et al. 2015; Petropoulos et al. 2016; Meistermann et al. 2021).

### Glucocorticoid exposure induces cell type–specific response

**Figure 2.**
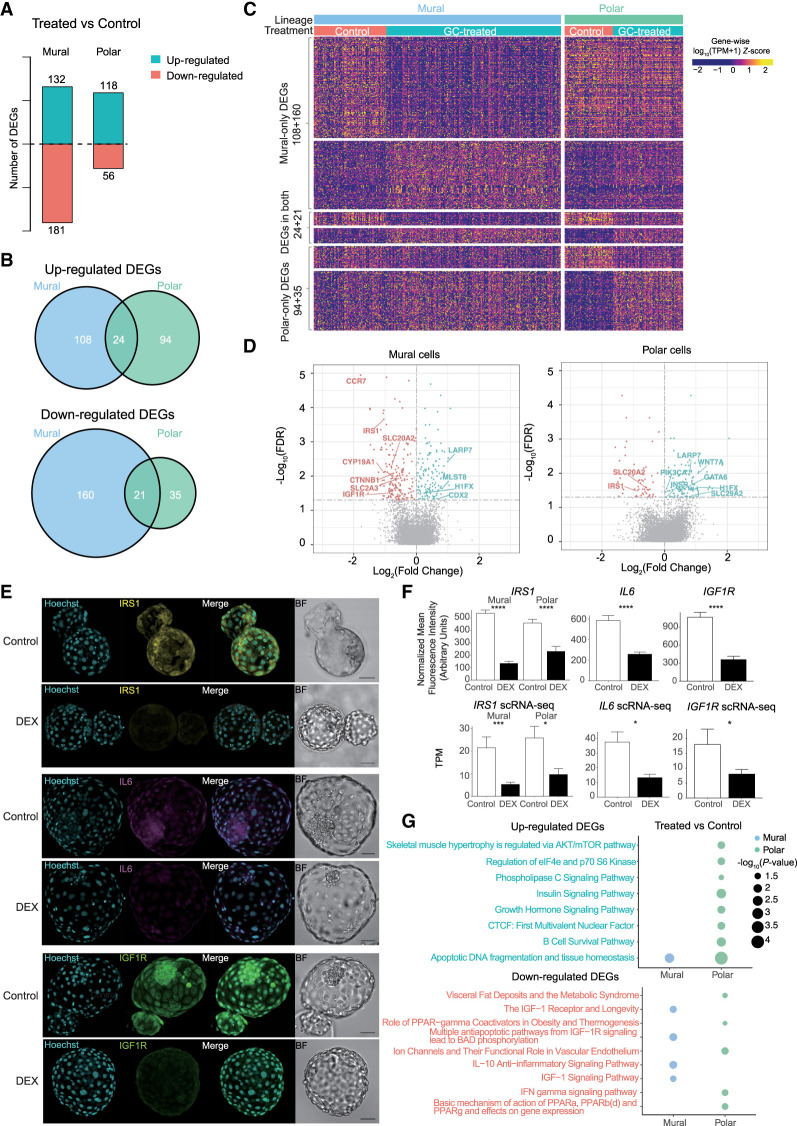
Glucocorticoid exposure induces cell type–specific responses. (*A*) The number of DEGs between control and glucocorticoid-treated embryos in mural and polar cells. The numbers *above* and *below* the *x*-axis represent the number of up-regulated and down-regulated DEGs in glucocorticoid-exposed embryos, respectively. (*B*) Venn diagrams of overlapping DEGs identified in mural cells and polar cells. (*C*) Heatmap showing the DEG expression level for the same groups as in the Venn diagrams in *B*. Color in the heatmap denotes the *z*-score of log-transformed TPM normalized expression values. (*D*) Volcano plot showing the expression fold change and the adjusted *P*-value (FDR) of treatment-related DEGs in mural and polar cells. The blue dots represent the up-regulated DEGs, and pink dots represent the down-regulated DEGs with glucocorticoid exposure. All significant DEGs associated with glucocorticoid exposure are found in Supplemental Table S3. (*E*) Validation-selected DEGs in the human embryo. Z-projection of immunofluorescence staining in E7 human embryos for IRS1, IL6, and IGF1R from control and DEX treatment (N = 5–7 embryos/treatment group). (BF) Bright field. Scale bars for all images are set at 50 µm. (*F*) Quantification of normalized mean fluorescence intensity and TPM values obtained from scRNA-seq. Significance: (****) *P* < 0.0001 using Student's *t*-test; (***) FDR < 0.001 and (*) FDR < 0.05 using “MAST” test for immunofluorescence and scRNA-seq, respectively. (*G*) Pathway enrichment analysis showing statistically significant BioCarta pathways following glucocorticoid exposure. The size of the circle represents the significance of pathways, and the color of the circle represents the lineages from which DEGs are identified.

Following cell assignment to lineage, we assessed the impact of glucocorticoid exposure on the global transcriptome in a lineage-specific manner to find treatment-related genes (Fig. 2). We performed differential expression analysis within each lineage between the treatment group and control (Fig. 2; Supplemental Fig. S3; Supplemental Table S3). This analysis identified 226 significantly up-regulated and 216 significantly down-regulated genes in the trophectoderm (TE) lineage between glucocorticoid-treated and control (Supplemental Table S3). We and others have previously shown that the TE lineage in the human embryo further specifies into a mural and polar sublineage starting at E6, and this segregation is owing to a differentiation process induced by the EPI, which becomes more pronounced at E7 (Petropoulos et al. 2016; Kagawa et al. 2022). In the embryo, it is the polar sublineage that initiates implantation into the uterine wall. Not surprisingly, the TE sublineages display a differential response to glucocorticoid treatment, which may be of importance given their varied biological roles in the embryo. Following treatment, 132 and 118 genes were up-regulated and 181 and 56 were down-regulated in the mural and polar sublineages, respectively (FDR≤5%) (Fig. 2A–C; Supplemental Fig. S3B; Supplemental Table S3). Further, 34 of the up-regulated and 18 of the down-regulated differentially expressed genes (DEGs) overlapped with the EPI and PE (Supplemental Fig. S3A; Supplemental Table S3). We focused all downstream analysis on the TE lineage given the abundance of literature available on the effects of glucocorticoid exposure in the placenta and trophoblast and the importance of the TE in establishing implantation. Among the significant DEGs between treatment and control, we found 24 genes were up-regulated in both the mural and polar sublineages, and 21 genes were down-regulated (Fig. 2B,C). The overlapping up-regulated genes included *LARP7*, known to be involved in RNA metabolism and noncoding RNA (Hasler et al. 2021), and down-regulated genes included *IRS1*, which mediates the effects of insulin and *IGF1* in the human preimplantation embryo (Lighten et al. 1997). From the 47 overlapping genes between mural and polar, only two genes displayed an opposite expression pattern, *MET* and *SNAR-C3* (Supplemental Table S3). Further, in the polar lineage, genes known to be markers of extravillous trophoblast, such as *WNT7A*, *EPSTI1*, and *B3GNT7*, were all significantly elevated following glucocorticoid treatment, suggesting that glucocorticoids can prematurely differentiate the human TE (Xiang et al. 2020).

### Impact of glucocorticoid treatment on gene-related functionality

To further examine the top candidate DEGs, we visualized the scRNA-seq with volcano plots. In the mural cells, several known glucocorticoid-responsive genes, such as *IGF1R*, *IRS1*, and *IL6*, which is known to cross talk with insulin/IGF1 signaling, were significantly down-regulated with treatment (Fig. 2D). This change in expression was validated with immunofluorescence and single-molecule fluorescence in situ hybridization (Fig. 2E,F; Supplemental Fig. S4A). Consistent with reduced insulin/IGF1 signaling, *MLST8*, a negative regulator of insulin/IGF1 signaling and component of mTOR signaling, was significantly up-regulated following glucocorticoid treatment (Fig. 2D). Further, polar lineage markers, *CYP19A1* and *CCR7* (Petropoulos et al. 2016; Meistermann et al. 2021), were down-regulated with treatment, whereas *CDX2* was up-regulated, further supporting the concept that glucocorticoids may play a role in differentiating the two TE sublineages. In polar cells, *IRS1* and *RXRA* were down-regulated and *INSR*, *GAL*, *LARP7*, *DPP4*, and *BATF* were significantly up-regulated following glucocorticoid exposure (Fig. 2D–F; Supplemental Fig. S4B) in addition to the expression of multiple members of the solute carrier family (e.g., *SLC20A2*, *SLC2A3*, *SLC38A2*) (Fig. 2D; Supplemental Table S3). However, expression of *IL6* and *IGF1R* was not significantly impacted in the polar lineage by glucocorticoid treatment, suggesting a lineage-specific response. Further, we leveraged the newly developed stem cell–based model for human preimplantation embryos, the blastoid (Kagawa et al. 2022). This model represents a promising new tool to study human preimplantation development and to catapult the fields of reproduction and development. The blastoid shows remarkable similarity to the human preimplantation embryo in terms of gene expression, timing of key developmental events, and ability to recapitulate aspects of implantation in vitro, using uterine cells (Zhao et al. 2021b; Kagawa et al. 2022). Here we wanted to assess the predictive potential of this embryo model by testing whether or not it responds to exogenous substrates, glucocorticoids, similarly to the human blastocyst. Analogous to the human embryo, blastoids were cultured in the presence of DEX or control and collected at 96 h, representing a time point equivalent to E7 human embryos (Supplemental Methods). Similar to what we observed in the human embryo following DEX treatment, there was a significant increase in BATF (*P* < 0.0001) in polar cells and a significant decrease in IL6 (*P* < 0.001) in mural cells (Supplemental Fig. S4A,B). This finding now provides an additional level for benchmarking human blastoids and supports a predictive value in studies involving drug screening, therapeutic interventions, and understanding fundamental aspects of human preimplantation development.

To explore the change in functionality associated with glucocorticoid exposure, we performed pathway enrichment analysis on all significant DEGs from E7 treatment versus control at a lineage-specific resolution (Fig. 2G; Supplemental Fig. S3D; Supplemental Table S3). Functional enrichment analysis revealed an increase in the insulin signaling pathway (*P* = 0.0034) and growth hormone signaling pathways (*P* = 0.014) in the polar cells and a down-regulation of IGF1 receptor and longevity (*P* = 0.022), multiple antiapoptotic pathways from IGF1R signal (*P* = 0.015), and IGF1 signaling pathway (*P* = 0.031) in the mural cells. In the polar cells, the top associated down-regulated pathways included visceral fat deposits and the metabolic syndrome (*P* = 0.043) and basic mechanism of action PPARa, PPARb(d) (*P* = 0.03). This analysis further emphasized that the TE sublineages display a differential response to glucocorticoid exposure and dysregulation of metabolic signaling and inflammatory response (Supplemental Table S3).

### Impact of glucocorticoid exposure on human preimplantation embryo methylome

DNA methylation plays a critical role in the regulation of gene expression and potential transmission of “reprogramming” marks. Given that glucocorticoids are known to alter DNA methylation, we wanted to determine whether this could serve as a potential mechanism underlying the transcriptional changes observed above. We sequenced the bisulfite-converted genomic DNA obtained from the same individual blastomeres as the mRNA using scM&T-seq (Angermueller et al. 2016). Blastomeres from 13 embryos were collected to explore the dynamics of the methylome among lineages (Supplemental Table S1). Following quality control, we had a total of 68 control and 46 treated cells with a minimum of 0.1 million (M) reads and 7% mapped reads (Supplemental Fig. S1C,D). The total number of covered CpGs and chromosome coverage was not biased by embryo or sex (Supplemental Fig. S5).

**Figure 3.**
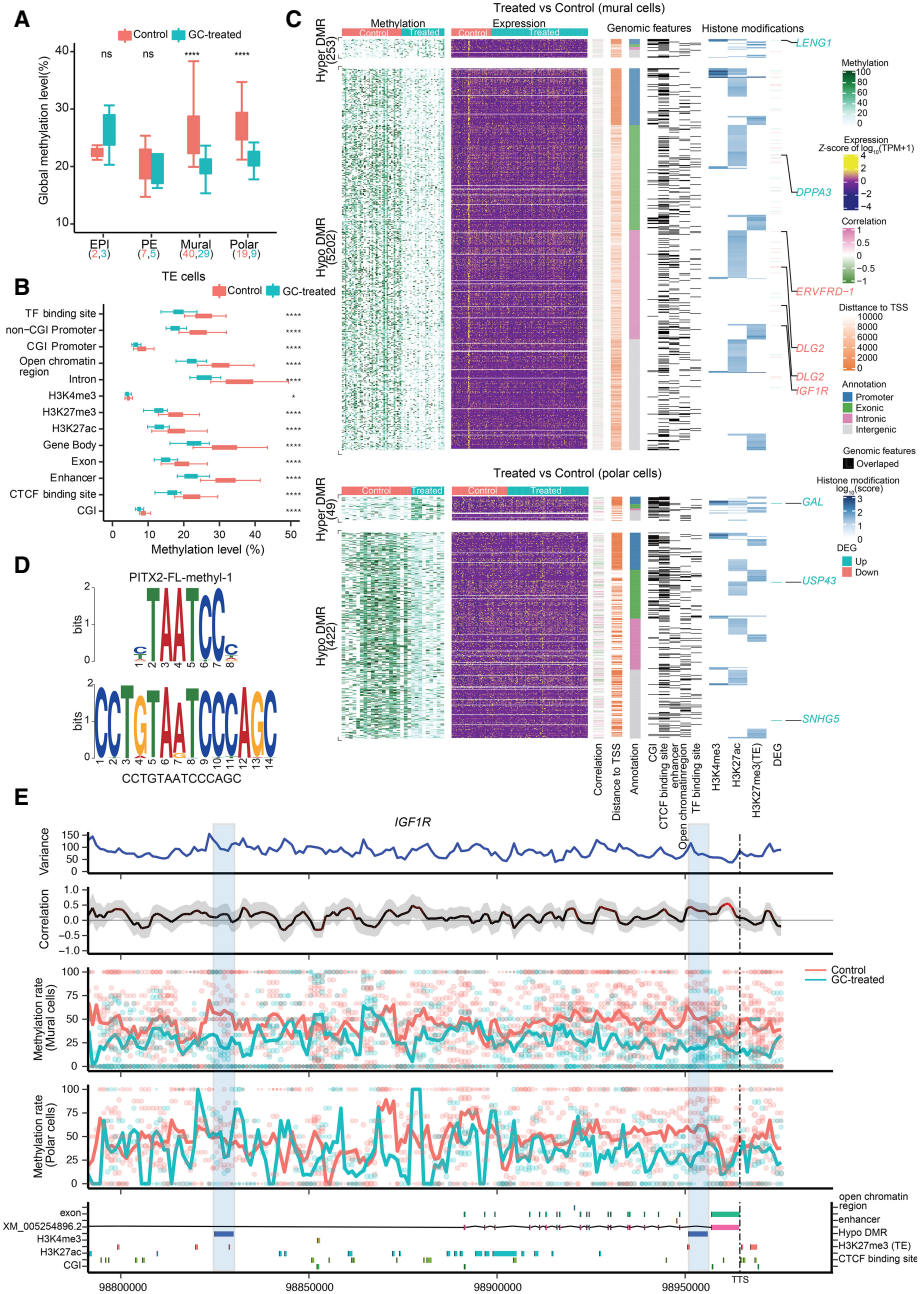
DMRs and genome-wide associations between methylation and transcriptional heterogeneity in control and glucocorticoid-exposed embryos. (*A*) Boxplot of the global DNA methylation levels showing the heterogeneity for each lineage in control and glucocorticoid-exposed embryos. The number of cells is labeled at the *bottom*. (*B*) Boxplot of methylation levels for different genomic contexts. (*C*) Comprehensive visualization of associations between methylation, expression, genomic features, and histone modification. From *left* to *right*, heatmap represents the methylation level of DMRs in individual cells with the number of DMRs indicated on the *left*. Missing values in DNA methylation heatmap are indicated with white: the expression level of nearest genes (from transcription start sites [TSSs], including different isoforms) for each DMR, the estimated weighted correlation between the DMRs’ methylation level and gene expression, the distance to its nearest TSS, the DMR annotation, the corresponding overlapping genomic features, the histone modification scores for the DMRs. Genes highlighted are the selective DEGs associated with the DMRs, where red represents down-regulated expression and blue represents up-regulated gene expression. (*D*) Top enriched sequence aligned to the most significant binding motif identified, PITX2 (217 hits, *P* = 1.2 × 10^−7^ and *P* of alignment = 0.0001). (*E*) Representative methylation variance, correlation, and methylation rate near the 3′ region of *IGF1R*. Shown from *bottom* to *top* are the annotation of the *IGF1R* locus with genomic features, histone modifications; the estimated methylation level of 3-kb sliding windows for each cell with dot size indicating CpG coverage, and dot colors indicating different treatments. The solid curve denotes the weighted mean methylation rate, with line colors representing different treatments and dashed vertical lines delineating the position of transcription termination sites (TTSs) of IGF1R. The correlation between the methylation rate and *IGF1R* expression for each window. Color of the curve represents the level of significance for the correlations, and the gray-shaded area denotes the 95% confidence interval of the correlation coefficient using the estimated weighted DNA-methylation variance between cells. Two hypomethylated DMRs identified between the control and treated mural cells are highlighted with blue rectangles.

We first leveraged the data from our control embryos to examine potential differences in methylation between the inner cell mass (ICM; containing the EPI and PE lineages) and TE lineage, as well as the mural and polar sublineages. In the control E7 embryos, we observed an average methylation level of 22.4%, 22.7%, 26.1%, and 27.8% for the EPI, PE, mural, and polar lineages, respectively, with median values displayed (Fig. 3A; Supplemental Table S1). This level of methylation is in line with that previously reported in E6 and E8 human embryos (Zhou et al. 2019). Moreover, using stringent parameters (see Supplemental Methods), we identified 217 differentially hypomethylated regions and 655 differentially hypermethylated regions between ICM and TE, and 126 differentially hypomethylated regions and 751 differentially hypermethylated regions between the mural and polar cells (Supplemental Table S4; Supplemental Figs. S6A, S7). When mural to polar cells were compared, 27 genes displayed significant DMRs and an associated correlation in gene expression, including polar lineage markers *TEAD3*, *KRT23*, and *IL1R1* (Supplemental Fig. S7; Supplemental Table S4; Petropoulos et al. 2016). The role of DNA methylation in mural–polar establishment would be of interest to further explore as studies examining mechanism(s) by which polar specification is achieved in the human embryo remain to be conducted.

When examining the 872 DMRs between the TE and ICM, we observed significant hypermethylation of *GATA3* (a TE lineage marker; CpG island [CGI]) and *SIN3B* (a histone deacetylase; promoter region), as well as hypomethylation of *DNMT3L* (a DNA methyltransferase; promoter region), *FGFBP3* (a fibroblast growth factor; promoter region), *SALL1* (involved in embryonic stem cell self-renewal; intergenic region), and *WNT7B* (involved in placental development; intergenic region). In total, 11 genes had significant DMRs correlated to a corresponding change in gene expression (Supplemental Fig. S6A; Supplemental Table S4), suggesting that these genes may play an important role in TE–ICM lineage segregation. *PTK2B* is one example in which a significant correlation between promoter methylation and gene expression is observed; a hypomethylated promoter corresponds to significantly up-regulated expression in ICM cells compared with the TE, regardless of treatment (Supplemental Fig. S6B). Further exploration of the genomic features and histone modifications located in this region (Chr 8: 27,324,000–27,329,000) revealed overlapping CpG islands with H3K27me3 histone modification, which suggests that multiple genomic-feature elements and epigenetic regulators synergistically regulate *PTK2B* gene expression.

Given that the mural and polar sublineages displayed a differential response to glucocorticoid exposure, we wanted to attain a more comprehensive overview of the relationship among DNA methylation, histone modification, and gene expression in these sublineages following treatment. Consistent with previous reports examining the impact of gestational glucocorticoids on the placenta (Crudo et al. 2012), we observed significant global hypomethylation (*P* < 0.01) (Fig. 3A) and hypomethylation at specific genomic regions (Fig. 3B) following treatment in both the mural and polar lineages. Overall, we observed 5202 and 422 significantly hypomethylated and 253 and 49 significantly hypermethylated DMRs in the mural and polar cells, respectively (Fig. 3C). These DMRs corresponded to 4159, 249, 409, and 49 genes, respectively (Supplemental Table S4). In the mural lineage, specific genes that were differentially methylated in response to treatment included promoter hypermethylation of *DNMT1* (a DNA methyltransferase) and *LENG1* (leucocyte receptor) and promoter hypomethylation of *AIRE* (a coordinator of immune tolerance and important for centrosome number regulation in the embryo) and *ERVV-1* (involved in the formation of syncytiotrophoblast cells). Hypermethylation in other genomic regions was also observed for *CYP11B1* (11-beta-hydroxylase enzyme; predicted CTFC_binding site), *DGCR8* (mediates biogenesis of miRNAs; predicted active H3K27ac and repressive H3K27me3 binding site), and the DNA methyltransferases *DNMT3A* and *DNMT3L* (at predicted enhancer region). In the polar lineage, we observed significant hypermethylation of *PLCB1* (involved in cell polarization in human embryos; promoter region) and hypermethylation of histone deacetylase *HDAC4* (histone deacetylase; intronic region) (Supplemental Table S4).

To identify transcription factors particularly influenced by DNA methylation, we performed a motif enrichment analysis for DMRs in promoter and distal elements (putative enhancer regions overlapping with H3K27ac or H3K27me3) (Supplemental Table S5; Fig. 3D) and compared the enriched sequences against a database containing motifs whose binding specificities are known to be influenced by DNA methylation (Yin et al. 2017). From this, we identified 45 significant motifs within the DMRs (Supplemental Table S5). Among the identified enriched sequences, significant predicted binding was associated with PITX2 (a core TE transcription factor, 217 hits; *P* = 1.2 × 10^−7^ and *P* of alignment = 0.0001) (Fig. 3D; Bai et al. 2012) and several nuclear receptors including RARs (63 sites; *P* = 0.016 and *P* of alignment = 0.00068), which elicit epigenetic control of stem cell differentiation and RXRB (63 sites; *P* = 0.016 and *P* of alignment = 0.00068), which results in DNA hypomethylation in cancer (Supplemental Table S5; Wang et al. 2019).

We next compared DEGs to DMRs and found distinct clustering of methylation and expression, suggesting that DNA methylation and transcriptome profiles yield complementary results for 52 specific genes, including *IGF1R*, following glucocorticoid treatment (Fig. 3C). We identified two hypomethylated regions associated with glucocorticoid exposure located on the second and last intron of *IGF1R,* both coinciding with H3K27me3 histone modification. Those two hypomethylated regions are mural specific and correspond to the differentially expressed *IGF1R* following glucocorticoid exposure (Fig. 3E).

### Glucocorticoid treatment precociously inactivates one X Chromosome

**Figure 4.**
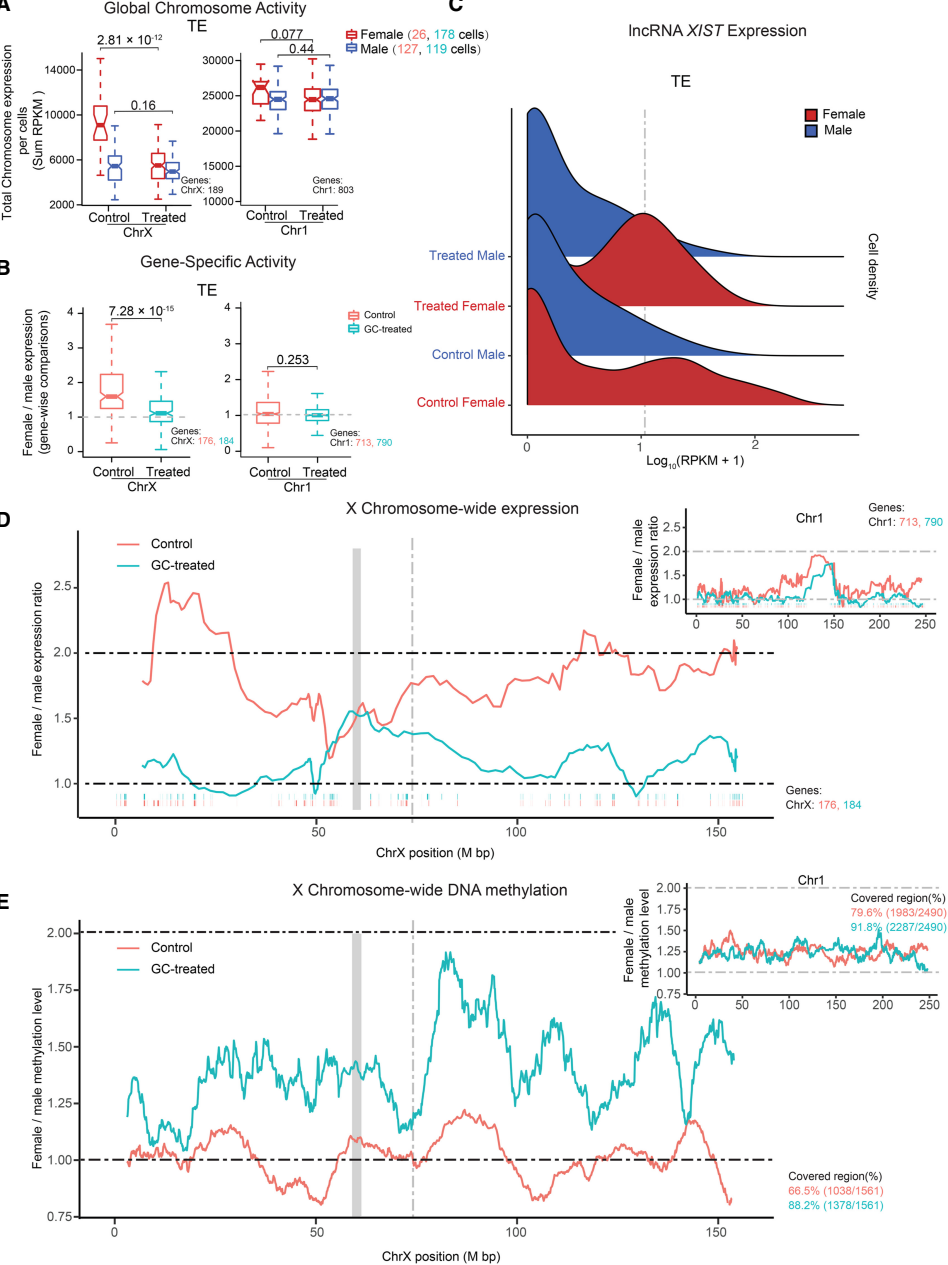
Glucocorticoid exposure perturbs X Chromosome dosage compensation. (*A*) Boxplots of X Chromosome and Chromosome 1 RPKM sums stratified by sex and treatment. Color represents sex. *P*-value was calculated using the two-sided Wilcoxon test. (*B*) Boxplots of female-to-male expression ratios of X Chromosome and Chromosome 1 linked genes in TE cells. Color represents treatment and control. *P*-value was calculated using the two-sided Wilcoxon test. (*C*) Ridge plot for *XIST* expression stratified by sex and treatment in TE cells. Color represents sex. (*D*) Sliding window (20-nearest genes) of female-to-male expression average along the X Chromosome for TE cells. The ticks *below* the moving-average lines indicate the location of expressed genes included in the estimates, colored according to different treatments. The gray block represents the locus of the centromere position. The gray dashed line denotes the locus of *XIST*. Similar analysis was performed for Chromosome 1. (*E*) Female-to-male moving methylation average along the X Chromosome in TE cells using a 50-nearest 100-kb sliding window. Chromosome 1 included for comparison. Colored according to different treatments. The gray block represents the locus of the centromere position. The gray dashed line denotes the locus of *XIST*.

Inactivation of the X Chromosome is important to achieve a dosage balance between males (XY) and females (XX). We and others have shown that in contrast to the traditional dogma of X Chromosome inactivation (XCI), in which one X Chromosome is inactivated, in the human preimplantation embryo and naive stem cells, a dual dosage compensation is observed in females, in which both X Chromosomes remain active (Schulz et al. 2014; Petropoulos et al. 2016; Vallot et al. 2017). How and why dual dosage compensation occurs in human embryos and when this is resolved to complete XCI remain largely unknown. To examine the impact of glucocorticoid treatment on X Chromosome activity, we first quantified the total X Chromosome output from male and female embryos in the TE lineage (mural and polar cells combined) (Fig. 4A) and separately for each sublineage (Supplemental Fig. S8A). We observed a significant decrease in global X Chromosome output only in female-treated cells (two-sided Wilcoxon test) (Fig. 4A, *P* = 2.81 × 10^−12^; Supplemental Fig. S8A, *P* = 5.13 × 10^−7^). Global expression output from Chromosome 1, serving as a control, and male cells remained unaltered. To further study the female-specific down-regulation of the X Chromosome, we calculated the female-to-male relative expression levels for specific X-linked genes (mean RPKM > 5) in the TE lineage (Fig. 4B) and separately for mural and polar (Supplemental Fig. S8B). Again, we observed a significant decrease in X Chromosome expression only in female-treated cells (two-sided Wilcoxon test) (Fig. 4B, *P* = 7.28 × 10^−15^; Supplemental Fig. S8A, *P* = 2.01 × 10^−14^). Together these analyses revealed that glucocorticoid treatment can significantly reduce X Chromosome activity in female TE cells.

The X-inactive species transcript (*XIST*) is located in the X inactivation center (XIC) and acts in *cis* to silence the X Chromosome (Brockdorff et al. 1991; Brown et al. 1992; Clemson et al. 1998). As expected, we observed higher *XIST* expression levels at E7 in the control female cells compared with males, whereas X Chromosome activity remained comparable (Fig. 4C; Petropoulos et al. 2016). In contrast, *XIST* expression was significantly increased in glucocorticoid-exposed female TE cells (*P* = 4.5 × 10^−9^ by two-sided Wilcoxon test based on RPKM values, female vs. male at E7) (Fig. 4C). This was also observed in both the mural and polar sublineages (Supplemental Fig. S8A–D). Next, we examined the methylation status of *XIST* following glucocorticoid treatment. Consistent with an increase in expression, we observed promoter hypomethylation and gene body hypermethylation (Supplemental Fig. S8E; Jones 2012). To investigate whether dampening of X Chromosome expression occurred chromosome-wide in the female embryo following glucocorticoid exposure, the female-to-male expression level was calculated by moving averages along the chromosome, revealing an X Chromosome–wide dosage compensation in response to glucocorticoid (Fig. 4D). Expression from autosomes, serving as negative controls, showed equivalent expression in control and treated cells (Fig. 4D). Using a similar approach as above, we calculated the female-to-male DNA methylation levels by moving averages of 100-kb windows along the chromosome. This analysis revealed an X Chromosome–wide hypermethylation in female-treated embryos (Fig. 4E), corresponding to the X Chromosome–wide decreased expression of X-linked genes. Autosomes, serving as negative controls, showed equivalent relative methylation levels in control and glucocorticoid-treated cells (Fig. 4E). Together, these data support our finding that glucocorticoid exposure results in premature XCI and provide evidence that this phenomenon is mediated, at least in part, by up-regulation of lncRNA *XIST* and X Chromosome–wide DNA hypermethylation.

### Small RNA expression in the human preimplantation embryo and the impact of glucocorticoid exposure

Small RNAs are known to regulate gene expression both transcriptionally and post-transcriptionally (Mattick and Makunin 2006; Matera et al. 2007). They are composed of multiple biotypes including miRNA, transfer RNA (tRNA), long intergenic noncoding RNA (lincRNA), small nuclear RNA (snRNA), and small nucleolar RNA (snoRNA). To date, the complete small RNA profile in the human embryo remains unknown. We therefore wanted to examine the small RNA biotypes in the TE control cells, as well as the impact of glucocorticoid on small RNAs. After quality control, we retained 154 cells (97 from control, 57 from treated) from eight embryos, with an average of 781 expressed small RNA and 53,081 small RNA molecules (including miRNA, tRNA, snRNA, and snoRNA) (Supplemental Figs. S1C,D, S9A,B; Supplemental Table S1). Generally, the length distribution and coverage of the small RNAs was as expected (Faridani et al. 2016; Hagemann-Jensen et al. 2018), confirming the integrity of our single-cell libraries (Supplemental Fig. S9A). Further, glucocorticoid treatment did not significantly influence either the size distribution or coverage of small RNA biotypes in the TE enriched cells (Supplemental Fig. S9A,B).

**Figure 5.**
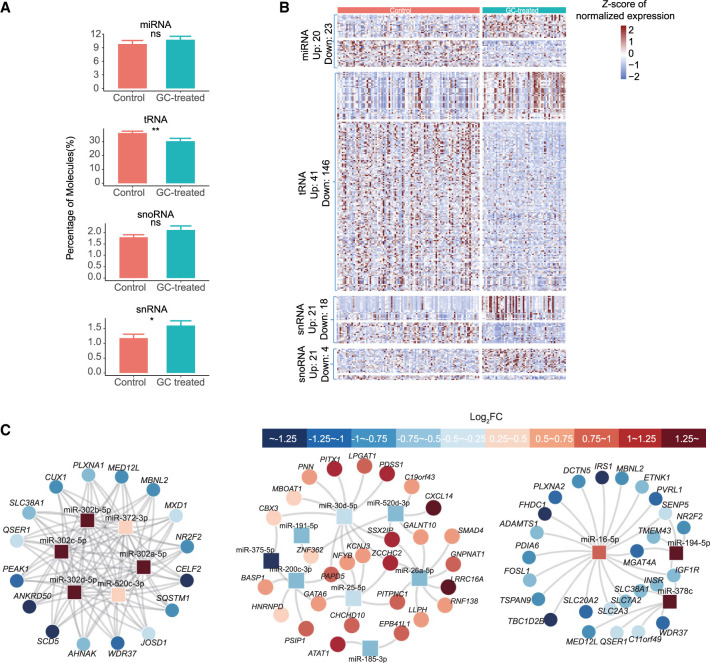
Characterization of small RNAs in control and glucocorticoid-exposed embryos. (*A*) The proportion of miRNAs, tRNAs, snoRNAs, and snRNAs in control and treated embryos. (*B*) Heatmap depicting the significant differentially expressed small RNA between control and glucocorticoid-exposed embryos. The type and the number of differentially expressed small RNAs are shown on the *left*. (*C*) Node-link diagram for differentially expressed miRNA–mRNA coexpression regulatory network. Red and blue represent log_2_ fold changes of expression between control and treated cells.

We confirmed the presence of all individual small RNA biotypes in the TE enriched cells (Fig. 5A). In control cells, the proportion of miRNA, tRNA, snoRNA, and snRNA was 9.83%, 36.3%, 1.79%, and 1.18%, respectively. Following glucocorticoid treatment, there was no significant impact on the global proportion of miRNA and snoRNA biotypes present (10.8% and 2.11%, respectively); however, there was a significant decrease in the number of tRNA molecules (30.4%) and a significant increase in the number of snRNA molecules (1.60%). To further explore the changes observed in small RNA biotype content in response to treatment, we performed differential expression analysis for each biotype. This analysis revealed 43, 187, 39, and 25 differentially expressed miRNAs, tRNAs, snRNAs, and snoRNAs, respectively (Fig. 5B; Supplemental Table S6). In comparison to miRNAs, far less is known about the other small RNA biotypes in the context of embryo development. Studies examining the precise functional role of these individual biotypes during embryogenesis are warranted and may contribute to our understanding of trophoblast stem cell populations, TE development, and placental development. The data generated here may serve as a resource for such studies.

In the context of development, miRNAs (18–25 nt) are the most widely studied. In mammals, miRNAs regulate ∼30% of protein-coding genes and are thereby able to regulate almost all biological processes (Nodine and Bartel 2010; Park and Kim 2013; Vidigal and Ventura 2015). To examine the potential regulatory role of differentially expressed miRNAs following glucocorticoid treatment, we generated miRNA–mRNA nodes. miRNAs significantly elevated in response to glucocorticoid (miR-302a-5p, miR-520c-3p, miR-302d-5p, miR-302c-5p, miR-372-3p, miR-9-5p, and miR-9-3p) target hormone-responsive genes such as *NR2F2* and *RXRA* (Fig. 5C; Supplemental Fig. S9C). Further, miRNAs significantly decreased in response to glucocorticoid (miR-25-5p, miR-200c-3p, miR-375-5p, and miR-26a-5p) target *SMAD4* (TE gene) and *NFYB* (regulates embryo cell maturation) (Fig. 5C; Salilew-Wondim et al. 2018). Three of the up-regulated miRNAs (miR-16-5p, miR-378c, and miR-194-5p) are associated with the *IRS1*, *IGF1R*, and several solute transporter genes (Fig. 5C), supporting the down-regulation in expression observed with treatment. Overall, these data suggest that the differential expression and dysregulation of insulin signaling observed in the TE following glucocorticoid treatment is mediated by DNA methylation and miRNAs.

## Discussion

Using a single-cell multi-omics approach, we have generated a comprehensive profile of the transcriptome, methylome, and small RNAs present in the human preimplantation blastocyst in control conditions and upon glucocorticoid exposure. These new data sets may serve as a resource for future studies aiming to further explore molecular determinants of embryo formation around the time of implantation. Further, we have elucidated the role of preimplantation glucocorticoid exposure and show its ability to precociously mature the TE lineage. This is supported by differentiation of the TE lineage, resulting in a more refined segregation between the mural and polar sublineages around the time of implantation and the presence of extravillous trophoblast markers in the polar cells, which more closely resembles the TE after implantation. In addition, we show that glucocorticoids have the ability to disrupt X Chromosome activity normally observed during this window of development and precociously initiate XCI. These findings may provide new insights into the regulation of XCI and human preimplantation development. Further, we have determined that glucocorticoid exposure during this window of development results in dysregulation of insulin and IGF1 signaling and an adaptive immune response in the TE, suggesting the potential development of altered placental capacity and metabolic disease later in life. Finally, leveraging the data generated by the untreated control cells, we generated the first comprehensive small RNA library in the human embryo, and via scM&T-seq, we have identified potential genes and DMRs that may be important for the ICM–TE and mural–polar lineage segregation.

The TE lineage gives rise to the placenta, the “life source” for the developing fetus. It is well known that dysregulation of placental growth and capacity to deliver nutrients to the developing fetus can result in both short-term and longer-term effects (Sferruzzi-Perri et al. 2017). Our data now suggest that glucocorticoid exposure of the preimplantation embryo leads to programmed molecular changes of solute transporters, insulin/IGF1 signaling, and immune response in the TE lineage and that these changes may result in suboptimal placental capacity and in turn longer-term adverse outcomes such as metabolic reprogramming of the offspring. The glucocorticoid-induced DEGs and associated pathways found in our study are in agreement with previous reports in animal studies showing that preimplantation glucocorticoid exposure results in altered metabolic programming (insulin signaling) and an adaptive immune response, related to modified expression of *IGF1*, *IRS1*, *INSR*, and *IL6* (Bronson and Bale 2014; Zheng et al. 2016). IGFs mediate their proliferative and antiapoptotic effects on trophoblast through activation of the IGF1R and possibly INSR triggering the MAPK and PI3K–AKT signaling pathways, respectively, therefore promoting placental growth (Diaz et al. 2007; Shields et al. 2007; Forbes et al. 2008; Mayama et al. 2013). Changes to the expression of insulin growth factors and their corresponding receptors in the placenta affect placental capacity and the ability to supply the fetus with nutrients, in turn impacting fetal growth (Sferruzzi-Perri et al. 2017). Further, in rats, protein deprivation reduces mTORC1 signaling, systems A and L amino acid transport, and *Slc38a2* expression in the placenta, before the appearance of fetal growth restriction (Jansson et al. 2006; Rosario et al. 2011). The preimplantation embryo has the ability to sense and adapt to its environment, so the decreased expression of *IGF1R, IRS1*, and solute transporters may be a protective adaptive response to minimize the effects of excess glucocorticoids. However, in doing so, the embryos may be inadvertently reprogrammed for metabolic dysregulation and disease development later in life. We acknowledge that the longer-term phenotype associated with these molecular changes is speculative, and further studies are warranted to determine the specific programming potential on human offspring. Animal studies examining the longer-term phenotype associated with preimplantation glucocorticoid exposure have shown numerous adverse outcomes, including altered metabolic health, cardiovascular health, and behavior (Mueller and Bale 2006; Burkuš et al. 2013; Rodgers et al. 2013; Bronson and Bale 2016; Jašarević et al. 2017; Chan et al. 2018). As such, it is not far-fetched to anticipate similar outcomes in humans.

Chromatin state and DNA methylation are important epigenetic modifications that can control transcription (Bird 2002). DNA methylation patterns are established by DNA methyltransferases DNMT3A and DNMT3B (de novo DNA methyltransferases) and are propagated by DNMT1, a maintenance methyltransferase (Suzuki and Bird 2008). During preimplantation development, there is a global wave of demethylation of parental genomes followed by a genome-wide remethylation of the embryonic and extraembryonic lineages around the time of implantation. Glucocorticoids are well-known modifiers of the epigenome, and indeed, we now show a global hypomethylated state in the embryo following exposure. These data suggest that glucocorticoids may interfere with the remethylation process that occurs in the late preimplantation embryo, which may be related to the altered expression and/or hypermethylation of key epigenetic factors *DNMT3A*, *DNMT1*, *H2AFV*, and *HDAC4*. Dysregulation of these “epigenetic factors” could result in a coordinated lack of de novo methyl marks and maintenance and altered nucleosome access. For instance, H2AFV is a “placeholder” nucleosome that restricts DNA methylation in zebrafish embryos (Madakashira et al. 2017). In our study, we observed a significant increase in *H2AFV* expression in mural cells and global hypomethylation following glucocorticoid treatment, suggesting that H2AFV may interfere with the DNA methylation marks that would normally be laid down. Targeted knockdown/inhibition, ChIP-seq, or experiments to assess chromatin state would be beneficial in determining the precise mechanism for the observed hypomethylation. Further, transcription factor binding site analysis predicted enrichment of PITX2 and several nuclear receptors, including retinoic acid receptor (RARB, RARG) and retinoic X receptor (RXRB), at DMRs overlapping with promoter or distal elements (putative enhancers and repressors, H3K27ac, H3K27me3, and H3K4me3). Retinoic acid and X receptors, RARs and RXRs, are ligand-activated transcription factors that interact with a large number of coactivators and corepressors to augment the epigenome and transcription (Wei 2003). Retinoid receptors modify the epigenetic landscape of embryonic stem cells during differentiation (Wei 2013) and induce DNA hypomethylation in cancer (Wang et al. 2019). These results suggest that specific transcription factors may have an adaptive response to glucocorticoid exposure that may act in concert with additional factors to form the epigenome and downstream phenotypes.

In addition to DNA methylation and chromatin-modifying enzymes, miRNAs are known epigenetic modulators. In our study, glucocorticoid treatment increased the expression of three miRNAs specifically related to *IGF1R*, *IRS1*, and *INSR* gene regulation: miR-16-5p, miR-378c, and miR-194-5p. The regulatory role of miR-16-5p and miR-194-5p on *IGF1R* expression has been previously confirmed and shown to result in altered proliferation of cells (Cheng et al. 2019; Bai et al. 2020). These data suggest that in addition to hypomethylation of *IGF1R* at an H3K27me3 site, increased expression of miRNAs targeting *IGF1R* may serve as a mechanism underlying the decreased expression observed in TE lineage.

We have previously shown in the human embryo that there is a dual dosage compensation for X Chromosomes that is present until E7 (Petropoulos et al. 2016). The developmental time and underlying mechanism for when this dual dosage is resolved in the female to achieve the traditional XCI remain unknown. We now show that glucocorticoid exposure can prematurely induce this resolution in the human preimplantation embryo and that this is, at least in part, owing to an X Chromosome–wide hypermethylation and simultaneous increase in expression of lncRNA *XIST*. This regulatory role of glucocorticoids on X Chromosome activity is novel and one that may be exploited to better understand XCI in humans or for the treatment of disorders associated with improper X Chromosome silencing. XCI initiation and maintenance involves multiple layers of molecular regulation including chromatin remodelers, PRC1 (establishes H2AK119ub mark) and PRC2 (establishes H3K27me3 mark). It would be of interest to further examine the impact of glucocorticoids on all genetic and epigenetic layers in order to better understand the role of glucocorticoids on XCI (Dixon-McDougall and Brown 2021, 2022; Loda et al. 2022).

In addition to the shedding light onto possible mechanism(s) underlying TE formation and XCI in the human embryo, this study serves as proof of principle that the human preimplantation embryo is susceptible to molecular reprogramming and that the first seven days following fertilization is a sensitive window of development. It also shows that human blastoids can be predictive of responses observed in human blastocysts, which opens possibilities to use blastoids to optimize IVF media and assess the reprotoxicity of drugs and chemicals. Currently, morphokinetics is the primary method used for assessing the effectiveness and safety of additives and adjuvants during ART. As shown in this study, despite the lack of change in morphology, blastocyst size, or cellular composition with treatment, profound molecular changes were observed at the level of gene expression, DNA methylation, and small RNAs. These findings highlight the need for prospective analyses of embryo cell health and a quantitative measure to assess the impact of specific additives and adjuvants. Given the rapid advances in technology, particularly the single-cell genomics field and the development of blastoids, which we have now shown respond to exogenous substrates similar to human embryos, these comprehensive molecular assessments are feasible. As a scientific community, we need to be more transparent with both the effectiveness and the possible consequences associated with the use of adjuvants and additives used in ART and the treatment of infertility. This will enable true informed consent from patients. We hope this study will serve as a proof of principle of the susceptibility of the human preimplantation embryo to exogenous exposures and open a larger conversation around the measures and endpoints used to assess the safety of adjuvants and additives in infertility.

## Methods

### Human embryo cultures for multi-omics sequencing

Human embryos were obtained from the Huddinge Karolinska Hospital with ethical approval from the regional ethics board (2018/691-31). Embryos were acquired fresh (not vitrified) from preimplantation genetic diagnosis (PGD) testing on E4 and were cultured until E7 (expanded blastocyst, just before implantation) under standard conditions as performed in the IVF clinic (5% CO_2_/5% O_2_ in IVF plates containing 700 μL of CCM media [Vitrolife] covered with 300 μL of Ovoil [Vitrolife] or in media supplemented with glucocorticoid [0.1 and 1 μg/mL DEX]) (González et al. 2010; Santana et al. 2014). DEX has 25–50 times the potency of the endogenous glucocorticoid, cortisol, but shares a similar structure (Magiakou and Chrousos 1997). A previous study using DEX (5–80 µg/mL) showed that only at doses of 10 µg/mL and above were negative effects observed on the morphokinetics of the embryo, including decreased blastocyst formation, expansion, and hatching (Van Merris et al. 2007). Blastocyst rates, total cell number, and embryo quality were endpoints evaluated on E7. All embryos used in this study showed normal morphology and developmental speed. Further, there was no visible difference in the number of cells allocated to each lineage (Supplemental Fig. S1A,B), similar to previous reports in the mouse in which embryos were cultured in the presence of DEX (González et al. 2010). Embryos were dissociated through trituration in TrypLE and handpicked with fine glass capillaries as previously described (Petropoulos et al. 2016). Cells were directly dispensed in a lysis buffer prepared according to the scM&T-seq protocol, and the cDNA libraries were generated using Smart-seq2 as previously described (Picelli et al. 2014; Angermueller et al. 2016; Petropoulos et al. 2016; Clark et al. 2017). Further, for a subset of embryos, cells were collected for Small-seq library preparation and data processing as previously described (Hagemann-Jensen et al. 2018). The quantity and quality of the cDNA, small RNA, and DNA methylation libraries were assessed using an Agilent 2100 Bioanalyzer. Indexed sequence libraries per molecular target were pooled for multiplexing and sequenced on Illumina HiSeq 2500 high output V4, SR 1 × 50 bp (scRNA-seq), PE 2 × 125 bp (DNA methylation) using a Nextera dual-index, iTag sequencing primers, and custom indexes for mRNA, DNA methylation, and small RNAs, respectively.

### scRNA-seq data preprocessing and quality control

For scRNA-seq data, the read quality was first checked by FastQC (v0.11.9) (https://www.bioinformatics.babraham.ac.uk/projects/fastqc/). High-quality reads were mapped to the human reference genome (build hg38, https://emea.support.illumina.com/sequencing/sequencing_software/igenome.html) using STAR (v2.5.3a) with default settings (Dobin et al. 2013), and only uniquely mapped reads were kept for gene expression quantification. Raw read counts and RPKM were estimated using rpkmforgenes (v1.0.1) (Ramsköld et al. 2009) with the option of “-readcount -fulltranscript -mRNAnorm -rmnameoverlap -bothendsceil” on transcripts derived from RefSeq annotation. We used library size, excluding mitochondrial genes, for computation of the transcripts per million mapped reads (TPM). Genes expressed (RPKM ≥ 1) in at least five cells were retained for analysis, giving 26,051 out of 33,097 genes. Cells were quality-filtered based on four criteria, leaving 505 cells after filtering out of 713 sequenced cells. First, library size without mitochondrial gene expression was required to be more than 50,000 reads. Second, the number of expressed genes per cell was required to be more than 3000. Third, based on the RPKM expression levels of all expressed genes, pair-wise Spearman's correlations between cells that passed the above criteria were calculated, and cells with maximum pair-wise correlations below 0.5 were filtered out. Fourth, cells belonging to embryo C6, D9, and D11 were removed because meiotic aneuploidies were detected (Supplemental Figs. S1C,D, S10; see section “Aneuploidy inference on scRNA-seq data” in Supplemental Methods). For detailed information and all downstream analysis, see Supplemental Methods.

### Inference of embryonic sex

The sex of each cell and embryo was inferred by the expression of Y-linked genes as we have previously described (Petropoulos et al. 2016). Cells with Y Chromosome RPKM sum (∑RPKM ChrY) > 100 were classified as male, and cells with ∑RPKM ChrY < 50 were classified as female. Embryo sex was determined by the ∑RPKM ChrY expressed in the majority of cells belonging to the same embryo. Cells with 50 < ∑RPKM ChrY < 100 and cells in conflict with other cells in an embryo were excluded from downstream X Chromosome analyses (Supplemental Fig. S1E,F). No sex differences are observed during the preimplantation stage for human embryos; therefore for all analyses, except for the XCI, we have collapsed the data for sex.

### Data integration, dimensionality reduction, and lineage segregation for scRNA-seq data

To identify the cell lineages from the scRNA-seq data, we integrated our data set, which was split into control and glucocorticoid-treated cells, with the Petropoulos et al. (2016) scRNA-seq data set, using only the day 7 embryos (Petropoulos et al. 2016). This resulted in three separate data sets. Normalization, scaling after regressing out the batch effect from embryos, and the number of expressed genes were performed separately on each of these data sets. Subsequently, all data sets were integrated using the canonical correlation analysis (CCA) approach implemented in the R package Seurat (v3.0.0) (Stuart et al. 2019) based on 12 dimensions and 500 anchor features. After integration, principal component analysis (PCA) was performed on the scaled data from the integrated object followed by embedding into low-dimensional space with UMAP and t-distributed stochastic neighbor embedding (t-SNE) as implemented by the RunUMAP() and RunTSNE() function. For clustering, the shared nearest neighbor (SNN) graph was constructed on the PCA embedding by calling the FindNeighbors() function followed by the identification of clusters using the FindClusters() function. Marker genes for each cluster were detected with the FindMarkers() function using the “roc” test under cutoff with the “power” more than 0.4, log fold-change more than 0.25, and >70% cells expressing the gene. Cell identity was inferred using our previously published lineage markers and cell annotation from Petropoulos et al. (2016; Fig. 1D,E; Supplemental Fig. S2A,B). Marker gene expression depicted in the heatmap represents the scaled *z-*scored residuals after regressing out the number of expressed genes and embryo by ScaleData() function. The above functions are all from the Seurat package (v3.0.0) (Stuart et al. 2019). The marker genes for each lineage and DEGs for each lineage comparison (control embryos) are listed in Supplemental Table S2.

### Differential gene expression analysis

Because of the difference in cell number obtained between mural and polar lineages, differential gene expression analysis was performed using MAST (v1.12.0) (Finak et al. 2015; Soneson and Robinson 2018) and TPM values to account for gene detection rate. For detailed information, see Supplemental Methods.

### Validation of DEGs by immunofluorescence

Human embryos were obtained from OVO Fertility with ethical approval from the regional ethics board (CERES 20.126). From E4 to E7, embryos were cultured as described above. For immunofluorescence validation experiments, human embryos were obtained, cultured, fixed, and stained as previously described (N = 5–7 embryos per antibody/treatment group) (Petropoulos et al. 2016). Primary antibodies used are as follows: mouse anti-IRS1 1:100 (Santa Cruz sc-8038), goat anti–IL6 1:100 (R&D AF-206-NA), mouse anti-BATF (Santa Cruz sc-1000974), and rabbit anti-IGF1R (Abcam ab182408). Secondary antibodies used were Alexa Fluor (Life Technologies) donkey anti-rabbit 488 conjugated 1:1000 (A-21206); donkey anti-goat 555 conjugated 1:1000 (A-21432); and donkey anti-mouse 647 conjugated 1:500 (A-31571). Images were acquired using an Olympus FV1000MPE confocal microscope equipped with a tunable Ti:Saf pulse laser 690- to 1040-nm multiphoton laser; 2-µm z-stacks were acquired with a XLUMPLFLN 20 × /1.00 WaterDipping objective and a zoom of 2.0. Images (dimension of 800 × 800 pixels) were acquired at 4 µs/pixel with the Fluoview software v. 4.2C and analyzed using ImageJ software version 1.53c. Normalized mean intensity values were obtained for individual cells with background subtracted to obtain arbitrary fluorescence values. An unpaired Student's *t*-test (*P* < 0.05) was performed in GraphPad Prism 9.2.0.

### DNA methylation data processing

Single-cell methylation reads were analyzed by the nf-core methylseq pipeline (v1.3) in bismark mode with presets for single-cell as previously described (Angermueller et al. 2016; Ewels et al. 2020). For detailed information and all downstream analysis, see Supplemental Methods.

### Motif enrichment analysis

Following identification of DMRs (Supplemental Methods), genomic sequences from DMRs that overlapped with either promoter or distal elements were analyzed for significant (*P*-value < 0.05) sequence motifs using simple, thorough, rapid, enriched motif elicitation (streme), a module of MEME suite (v5.4.1) (Bailey et al. 2015) with default parameters. Distal elements included enhancer regions, as determined using regulatory features annotated in the Ensembl database, and enhancer regions that also overlapped with either H3K27ac or H3K27me3. The identification of significant sequence motifs was followed by the Tomtom module of MEME suite with default parameters. This allowed us to compare the significantly enriched motifs against a database of known motifs for which methylation is known to influence DNA binding specificities of human transcription factors (Yin et al. 2017). PITX2 was identified as the most-likely aligned motif (ranked by *P*-value), Figure 3D. A comprehensive list of significant motifs can be viewed in Supplemental Table S5.

### X Chromosome expression and methylation analysis

X Chromosome expression analysis was conducted as we have previously described, with some modifications (Petropoulos et al. 2016). For detailed information, see Supplemental Methods. X Chromosome methylation analysis was calculated in a similar manner. Briefly, the X Chromosome was tiled into 100-kb non-overlapped bins. Then the methylation level for those 100-kb bins in individual cells was calculated as in the section “DNA methylation data processing.” Subsequently, 100-kb bins with read coverage greater than 10 in at least five female and five male cells were selected. The mean methylation level was calculated for female and male cells separately, followed by a female-to-male ratio for each 100-kb bin to obtain fold change. The moving average of female-to-male relative methylation shown in Figure 4E was conducted using the mean ratio from the sliding window of 50 bins. Methylation level of *XIST* promoter region (regulatory feature, ENSR00000247242) and gene body were calculated by the weighted mean methylation level of all female TE cells stratified by treatment (Supplemental Fig. S8E).

### Small RNA processing

Small RNA reads were processed by the Small-seq pipeline as previously described (Faridani et al. 2016; Hagemann-Jensen et al. 2018). For detailed information and all downstream analysis, see Supplemental Methods.

### miRNA–mRNA network analysis

The miRNA target networks were determined by miRTarVis software (L'Yi et al. 2017) using the following steps. First, FDR and log_2_ (fold change) values of differentially expressed miRNAs and genes between different treatments in mural or polar cells with at least 0.25 log_2_ (fold change) were loaded into miRTarVis. Fold change of mRNA was calculated using the mean expression for TE cells from control and glucocorticoid-treated embryos. Second, only miRNA–mRNA pairs were used with opposite differentially expressed direction from TargetScan. Third, “KK-layout” was selected for visualization followed by image export.

### Data access

All raw and processed sequencing data generated in this study have been submitted to ArrayExpress (https://www.ebi.ac.uk/arrayexpress/) under accession numbers E-MTAB-10096, E-MTAB-10097, and E-MTAB-10098.

## Supplementary Material

Supplemental Material
