# Supplementary material for: Single-cell multi-omics of human preimplantation embryos shows susceptibility to glucocorticoids
Source: Genome Res. 2022 Sep;32(9):1627–41. doi: 10.1101/gr.276665.122 (PMC9528977; doi:10.1101/gr.276665.122)
Supplement: Supplemental Material [file supp_32_9_1627__DC1.html]

Single-cell multi-omics of human preimplantation embryos shows susceptibility to glucocorticoids — Supplemental Material 

# Single-cell multi-omics of human preimplantation embryos shows susceptibility to glucocorticoids

## Supplemental Material

- Supplemental\_Material.docx
- Supplemental\_Table\_S1.xls
- Supplemental\_Table\_S2.xls
- Supplemental\_Table\_S3.xls
- Supplemental\_Table\_S4.xls
- Supplemental\_Table\_S5.xls
- Supplemental\_Table\_S6.xls
