# Supplementary material for: Single-cell multi-omics of human preimplantation embryos shows susceptibility to glucocorticoids
Source: Genome Res. 2022 Sep;32(9):1627–41. doi: 10.1101/gr.276665.122 (PMC9528977; doi:10.1101/gr.276665.122)
Supplement: Supplemental Material [file supp_gr.276665.122_Supplemental_Material_.docx]

Supporting Online Material for:

​​***Single-cell multi-omics of human preimplantation embryos demonstrates susceptibility to glucocorticoids***

Cheng Zhao^1^, Savana Biondic^2,3^, Katherine Vandal^2,3^, Åsa K Björklund^4^, Michael Hagemann-Jensen^5^, Theresa Maria Sommer^6^, Jesica Canizo^2,3^, Stephen Clark^7,8^, Pascal Raymond^9^, Daniel R. Zenklusen^9^, Nicolas Rivron^6^, Wolf Reik^7,8,10,11^, Sophie Petropoulos^1-3#^

***To whom correspondence should be addressed.**

**Email:** sophie.petropoulos@ki.se, [sophie.petropoulos@umontreal.ca](mailto:sophie.petropoulos@umontreal.ca)

**This PDF file includes: Supplemental Methods and Supplemental Fig. S1-S10.**

**Supplemental Methods**

**Immunostaining and imaging of human embryos**

Human embryos were obtained from OVO Fertility with ethical approval from the regional ethics board (CERES 20.126). From E4-E7, embryos were cultured under standard conditions (control) or media supplemented with glucocorticoid (DEX, 0.1 μg/ml and 1μg/ml) (González et al. 2010; Santana et al. 2014). Embryos were fixed and stained as previously described (Petropoulos et al. 2016). For nuclear staining, Hoechst (33342, Invitrogen) was diluted in blocking solution and embryos were stained, after secondary antibody incubation, for 10 min at room temperature. Embryos were mounted in PBS and placed between two cover glasses using silicon spacers (Grace Bio-labs). For morphological assessment, primary antibodies used: rabbit anti-NANOG 1:200 (500-P236, Peprotech); goat anti-GATA6 1:100 (AF1700, R&D); mouse anti-CDX2 1:250 (MU392A, Biogenex). Secondary antibodies used were Alexa Fluor (Life Technologies) donkey anti-rabbit 488 conjugated 1:1000; donkey anti-goat 555 conjugated 1:1000; donkey anti-mouse 647 conjugated 1:1000. Images were acquired using an Olympus FV1000MPE confocal microscope equipped with a tunable Ti:Saf pulse laser 690-1040 nm multi-photon laser, 2 µm z-stacks were acquired with a XLUMPLFLN 20×/1.00 WaterDipping objective and a zoom of 2.0. Images (dimension of 800 × 800 pixels), were acquired at 4 µs/pixel with the Fluoview software v. 4.2C and analyzed using ImageJ software version 1.53c. All embryos developed to blastocysts, with a distinct ICM (epiblast (EPI) and primitive endoderm (PE). Measurements we made to assess total cell count, number of cells per lineage and blastocyst diameter (Supplemental Fig. S1A,B). Statistics and bar graphs were done in Graphpad Prism9.2.0. Given the lack of differences following developmental parameters, we chose 1μg/ml dexamethasone to perform downstream multi-omics experiments.

**Human blastoid culture**

H9 naive pluripotent stem cells (PSCs) were used for blastoid formation. Naive cells were cultured routinely on gamma-irradiated mouse embryonic fibroblasts (MEFs) in PXGL medium (Guo et al. 2017) and human Blastoids were formed as reported previously (Kagawa et al. 2022). In brief, naive embryonic stem cells were incubated with Accutase (Biozym, B423201) at 37 °C for 5 min, dissociated into single cells and spun down. Cells were resuspended in PXGL medium containing 10 µM Y-27632 (MedChemExpress, HY-10583) and transferred onto gelatin coated 6-well plates for 70 min at 37°C. Thereafter, cells were collected, and the cell number was determined using a Countess automated cell counter (Thermo Fisher Scientific). Cells were spun down and resuspended in N2B27 supplemented with 10 µM Y-27632. A total of 3.0 × 10^4^ cells were seeded onto a microwell array into a well of a 96-well plate and placed in a hypoxic chamber (5% CO2/5% O2) for 24 h. Subsequently, the aggregation medium was removed and 150 µl of PALLY medium - N2B27 medium supplemented with PD0325901 (1 µM, MedChemExpress, HY-10254), A 83-01 (1 µM, MedChemExpress, HY-10432), 1-oleoyl lysophosphatidic acid sodium salt (500 nM, Tocris, 3854), hLIF (10 ng/ml, in house) and Y-27632 (10 µM)) was added. PALLY was exchanged after 24 h. For the following media change, blastoids were either cultured in PALLY or in PALLY supplemented with glucocorticoid (1 μg/ml, DEX). After 48 h, medium was exchanged to N2B27 medium with 500 nM LPA and 10 µM Y-27632 (LY medium) or LY medium with glucocorticoid. After 96 h fully developed blastoids were selected for downstream analysis.

**Validation of differentially expressed genes in blastoids**

For immunofluorescence validation experiments mature human blastoids were fixed in 1% Paraformaldehyde in PBS for 30 min (N=4-9 blastoids per antibody/treatment group). Formaldehyde was removed, and samples were washed 3 times with PBS. Blastoids were permeabilized and blocked using 0.3% Triton X-100 and 10% donkey serum in PBS for at least 60 min. Primary antibodies were diluted in blocking/permeabilization solution as followed: goat anti–IL6 1:100 (AF-206-NA, R&D), mouse anti-BATF (sc-1000974, Santa Cruz). Samples were incubated with primary antibodies at 4°C overnight. The following day, blastoids were washed with PBS containing 0.1% Triton X-100 (PBST) three times for a minimum of 10 min each. Secondary antibodies and Hoechst nuclear stain were diluted 1:300 in blocking/permeabilization solution and added to the samples for 1 hour. Subsequently, samples were washed three times for at least 10 min. Blastoids were mounted on µ-Slide Angiogenesis glass bottom slides (Ibidi, 81507) in PBS. Human Blastoids were imaged on an Olympus IX83 microscope with Yokogawa W1 spinning disk (Software: CellSense 2.3; camera: Hamamatsu Orca Flash 4.0). Z-stacks were acquired using 2µm step size with a 20×/0.75 Air objective. Images were analysis in ImageJ, version 1.53f51. Unpaired Student’s *t*-test (*P*<0.05) was performed in Graphpad Prism9.2.0.

**Single molecule fluorescence *in situ* hybridization (smFISH)** **in human embryos**

smFISH was performed as we previously described (Petropoulos et al. 2016). Briefly, samples were hybridized for 6 h at 38.5°C in a humidity chamber with *IL6* CAL Fluor Red 610 (VSMF-20534-5; BioSearch Technologies) then mounted with Prolong Diamond antifade (ThermoFisher Scientific) and allowed to dry for 24 h in the dark at room temperature before imaging. Samples were imaged using a Zeiss Axio-Imager Z2 upright microscope with 63× 1.4 NA oil-immersion objective, Lumen Dynamics X-Cite illuminator, Photometrics sCMOS 16 bit camera and and Chroma SP103v1 Cy3.5 filter, with 0.24 µm z-stack step size. Unpaired Student’s *t*-test (*P*<0.05) was performed in Graphpad Prism9.2.0.

**Aneuploidy inference on scRNA-seq data**

Chromosomal aberrations were checked by inferCNV (v1.3.6) of the Trinity CTAT Project (Tickle et al. 2019) using raw gene expression as recommended. For the inferCNV analysis the following parameters were used: “denoise”, default hidden markov model (HMM) settings, and a value of 1 for “cutoff”, “NULL” for “ref_group_names”. Subsequently, copy number values (CNV) per gene were extracted from “run.final.infercnv_obj” and the ploidy state for each was further inferred by the majority type of the genes which were determined as “gain”(CNV >1.25), “loss” (CNV <0.75) and “normal”( 0.75 ≤ CNV ≤ 1.25) (Supplemental Fig. S10A). To confirm the CNV results inferred from scRNA-seq data, we also checked the read coverage from the paralleled methylation data. The total single-cell bisulfite sequencing reads of each 10 million (M) base-pair (bp) window were calculated and normalized using the total mapped reads of each sample. For each 10M bp window, the normalized coverage was further corrected by dividing the average normalized coverage across all samples to rule out regional sequencing bias (Supplemental Fig. S10B,C). For embryos C6, D9 and D11, a high proportion of aneuploid cells (53.3%, 45.8% and 82.1%) were observed after quality control. Although it has been reported that differentiation of the major lineages are not distorted by slight CNVs at the early stage of implantation (Zhou et al. 2019), we excluded all sequencing data for those three embryos to avoid over/underestimating the impact of glucocorticoid exposure.

**Differential gene expression analysis**

Due to the difference in cell number amongst mural and polar lineages, differential gene expression analysis was performed using MAST (v1.12.0) (Soneson and Robinson 2018; Finak et al. 2015), using TPM values to account for gene detection rate. Genes with an FDR less than 0.05 and expressed in more than 33% of cells were considered to be differentially expressed. Given the low number of cells in the epiblast and primitive endoderm lineages, differential gene expression analysis between control and treated cells was performed using the ‘roc’ test with a “power“ of >0.6 and requirement for genes to be expressed in more than 33% of compared cells. Sex chromosome genes were filtered out to avoid influence from uneven distribution of embryo sex between control and treatment. Expression data for differentially expressed genes depicted in heatmaps ﻿are *z*-scored scaled, log-transformed TPM values. Raw counts for cells belonging to the same embryo with more than 6 cells in mural or polar lineages, were aggregated into a pseudo-bulk sample. Normalization of expression for pseudo-bulk samples was calculated by estimateSizeFactors function, followed by estimateDispersions function from R package DESeq (Anders and Huber) (v1.38.0). The log-transformed and normalized gene expression values of DEGs between control and glucocorticoid-treated embryos in mural and polar cells were utilized to generate the heatmap in Supplemental Fig.S3B. The Gene Ontology (GO) term enrichment analysis for differentially expressed genes was conducted using the R package TopGO (v2.38.1) (Alexa and Rahnenfuhrer 2016) utilizing their improved weighted scoring algorithm and Fisher’s test to define the significance of GO term enrichment. Functional enrichment analyses were performed using the R package ClusterProfile (v3.14.3) (Yu et al. 2012). BioCarta functional annotations were downloaded from EnrichR database (Kuleshov et al. 2016; Rouillard et al. 2016). Significantly enriched GO and functional terms were identified as those with a *P*-value less than 0.05.

**DNA methylation data processing**

Single-cell methylation reads were analyzed by the nf-core methylseq pipeline (v1.3) in bismark mode with presets for single-cell as previously described (Ewels et al. 2020; Angermueller et al. 2016). Raw reads were trimmed to remove the first 6 bp (the 6N random priming portion of the reads), adaptor contamination and low quality base calls using Trim Galore (v0.5.0) (parameters: --clip_r1 6 --clip_r2 6 --three_prime_clip_r1 6 --three_prime_clip_r2 6) (Krueger 2015). Subsequently, the clean reads were mapped to the human genome (build hg38) by Bismark (v0.20.0) (parameters: –non_directional) which was followed by de-duplication and DNA methylation calling by deduplicate_bismark and bismark_methylation_extractor from Bismark tool with default settings (Krueger and Andrews 2011). 114 out of 280 cells were kept after quality filtering based on two criteria. First, cells that failed in the RNA-Seq QC processing were removed. Second, cells with a low alignment rate (alignment rate < 7%) or a low library size (<0.1 million (M) reads) were filtered out. The methylation rate for each region of interest (e.g. genomic features, regions with histone modification, 3 kb sliding windows, differentially methylated regions, and 100 kb bins) was estimated as the mean CpG methylation rate for the corresponding region in individual cells. The methylation level of CpG di-nucleotides was estimated from the ratio of methylated read counts to total read counts. No obvious sequencing bias by the distribution of embryo and sex were observed for this dataset (Supplemental Fig. S5).

**Correlation analysis between gene expression heterogeneity and DNA-methylation heterogeneity**

Correlation analysis across cells was conducted as previously described (Angermueller et al. 2016; Clark et al. 2018). Briefly, selected loci were required to be covered by at least one read in at least 10% cells. All possible relationships between genes and methylated regions within 10 kb of the gene were considered in accordance with the original protocol papers (Angermueller et al. 2016). Association tests were based on the weighted Pearson correlation coefficient between log-transformed TPM values and DNA methylation level of test regions considering the differences in CpG coverage across cells. Two-sided Student’s *t*-tests were performed to test for nonzero correlation, and *P*-values were adjusted by Benjamini-Hochberg for each context. For the gene-wise plot in Fig. 3E and Supplemental Fig. S6B, the methylation level of 3 kb sliding windows with a step size of 1 kb within 10 kb of the gene were estimated and tested for association with gene expression. Weighted correlation coefficients were calculated in the same way as defined above. Analysis for calculating the weighted correlation between methylation level of differentially methylated regions (DMRs) and expression of the gene that has the nearest TSS site to the DMRs were similarly performed as described above. The heatmap represents the methylation level of DMRs and corresponding gene expression level was performed using the R package ComplexHeatmap (v2.2.0 ) (Gu et al. 2016).

**Identification of differentially methylated regions**

For each analysis performed, we treated coverage of every CpG site as the number of cells with coverage at that site and treated methylation fraction as the fraction of cells that had a methylated CpG at that site. A methylated CpG site was defined as the CpG site with methylation level more than 0.5. Only cytosines with coverage in both control and glucocorticoid-treated were taken into account and the cytosine methylation level for every 3 kb window, with a step size of 1 kb, was compared using Fisher’s exact test. Windows with an adjusted *P*-value (corrected by Benjamini–Hochberg method) less than 0.05, more than 2-fold change in DNA methylation level, and two or more differentially methylated cytosines (DMCs, defined as cytosines with *P*-value < 0.05 in Fisher’s exact test) were further retained and merged to generate DMRs. Finally, DMRs with no coverage in less than two cells for each compared group were discarded. DMRs were annotated using the R package ChipSeeker (v1.22.1) (Yu et al. 2015).

**Genomic features and histone modification**

Regions for CTCF binding sites, transcription factor binding sites, enhancers, and open chromatin regions were extracted using the regulatory features annotation of Ensemble database (release-97) (Zerbino et al. 2015). CpG island (CGI) annotation was downloaded from UCSC Genome Browser (golden path for hg38) (Haeussler et al. 2019). “CGI Promoter” was defined as the upstream 3 kb window from the gene transcriptional start site overlapping with at least one CGI. “non-CGI Promoter” was similarly determined but containing no overlapping with CGI. Processed data of H3K4me3, H3K27me3, and H3K27ac were downloaded from GSE124718 (Xia et al. 2019) and converted to hg38 coordinates using liftOver tool (Haeussler et al. 2019). H3K27ac and H3K4me3 modifications were estimated from inner cell mass cells (ICM) only. H3K27me3 were estimated from both ICM and trophectoderm (TE) cells and combined when cell type was unspecified. Histone modification score is from the 5th column of processed data according to UCSC broadPeak description. The score was averaged if a DMR overlapped with multiple histone modification regions.

**X Chromosome expression and methylation analysis**

X Chromosome expression analysis was conducted as we have previously described, with some modifications (Petropoulos et al. 2016). Briefly, the RPKM sums for X Chromosome shown in Fig. 4A were calculated per cell using genes with 5<mean RPKM<200 calculated over cells stratified by lineage. The upper threshold was applied to prevent the weight of a few highly-expressed genes from dominating the estimate. RPKM was used instead of TPM to avoid the influence of different transcript lengths. To calculate the gene-wise female-to-male fold-changes shown in Fig. 4B,D, genes were first selected with the mean RPKM values, calculated over the cells stratified by treatments, lineage, sex and with RPKM>5 in both female and male cells. For individual genes, mean RPKM values were calculated for female and male cells separately and expressed as a female-to-male ratio to obtain fold change. The moving average of female-to-male relative expression shown in Fig. 4D was conducted using the mean ratio from the sliding window of 20 genes. X Chromosome methylation analysis was calculated in a similar manner. Briefly, the X Chromosome was tiled into 100 kb non-overlapped bins. Then the methylation level for those 100 kb bins in individual cells was calculated as in the section of “DNA Methylation Data Processing”. Subsequently, 100 kb bins with read coverage >10 in at least five female and five male cells were selected. Mean methylation level was calculated for female and male cells separately, followed by a female-to-male ratio for each 100 kb bin to obtain fold change. The moving average of female-to-male relative methylation shown in Fig. 4E was conducted using the mean ratio from the sliding window of 50 bins. Methylation level of *XIST* promoter region (Regulatory Feature: ENSR00000247242) and gene body were calculated by the weighted mean methylation level of all female TE cells stratified by treatment, Supplemental Fig. 8E.

**Small RNA processing**

Briefly, reads aligning over the full sequence (66 nucleotides (nts); our maximum sequence length for small RNA-Seq was 76 bp and required the removal of eight bases associated with UMIs and the ‘CA’ bases) were assigned as precursor molecules, whereas reads aligning with 65 nts or less were assigned as a potential small ncRNA. The reads corresponding to the 8bp associated with the UMIs were removed from sequence reads and appended to the reads’ name using umi_tools (v0.4.4) (Smith et al. 2017), followed by cutadapt (v1.17) (Martin 2011) (parameters: -e 0.1 -O 1 -m 18 -u 2) to remove the adapter and poly(A) sequences. Subsequently, clean reads were aligned to the human genome (build hg38) using STAR with the following parameters “--outSAMstrandField intronMotif –outFilterMultimapNmax 50 –outFilterScoreMinOverLread 0 –outFilterMatchNmin 18 –outFilterMatchNminOverLread 0 –outFilterMismatchNoverLmax 0.04 –alignIntronMax 1”. Insertion, deletion or soft-clipping mapping up to 3 nts at the 3’ end of the reads were removed. PCR amplicons were collapsed and RNA molecules were counted using UMI-tools with the option of “dedup --method adjacency”. The expression of miRNA (Mirbase V22) (Kozomara et al. 2019), tRNA (GtRNAdb hg38) (Chan and Lowe 2016), snRNA and snoRNA (Gencode V29) (Harrow et al. 2012) were estimated by count_smallrnas.py from the published Small-seq pipeline (Hagemann-Jensen et al. 2018; Faridani et al. 2016). Cells were then filtered based on two criteria: First, the proportion of mitochondrial associated UMIs was required to be less than 20%. Second, the number of expressed small ncRNAs (miRNA, tRNA, snRNA and snoRNA) per cell was required to be more than 300. Following quality-control, 154 cells (out of 192 sequenced) were retained for downstream analysis.

**Normalization and differential expression analysis for small RNA**

To minimize the difference in sequencing depth, UMI count of small RNA was normalized for each biotype by running the vst() function as implemented in the R package sctransform (v0.2.1) (Hafemeister and Satija 2019). Differential expression for small RNA was performed by running the compare_expression() function found in the stransform package. Small RNAs with FDR less than 0.05 and expression in more than 33% of cells were considered to be differentially expressed.

**Supplemental Figures**

**
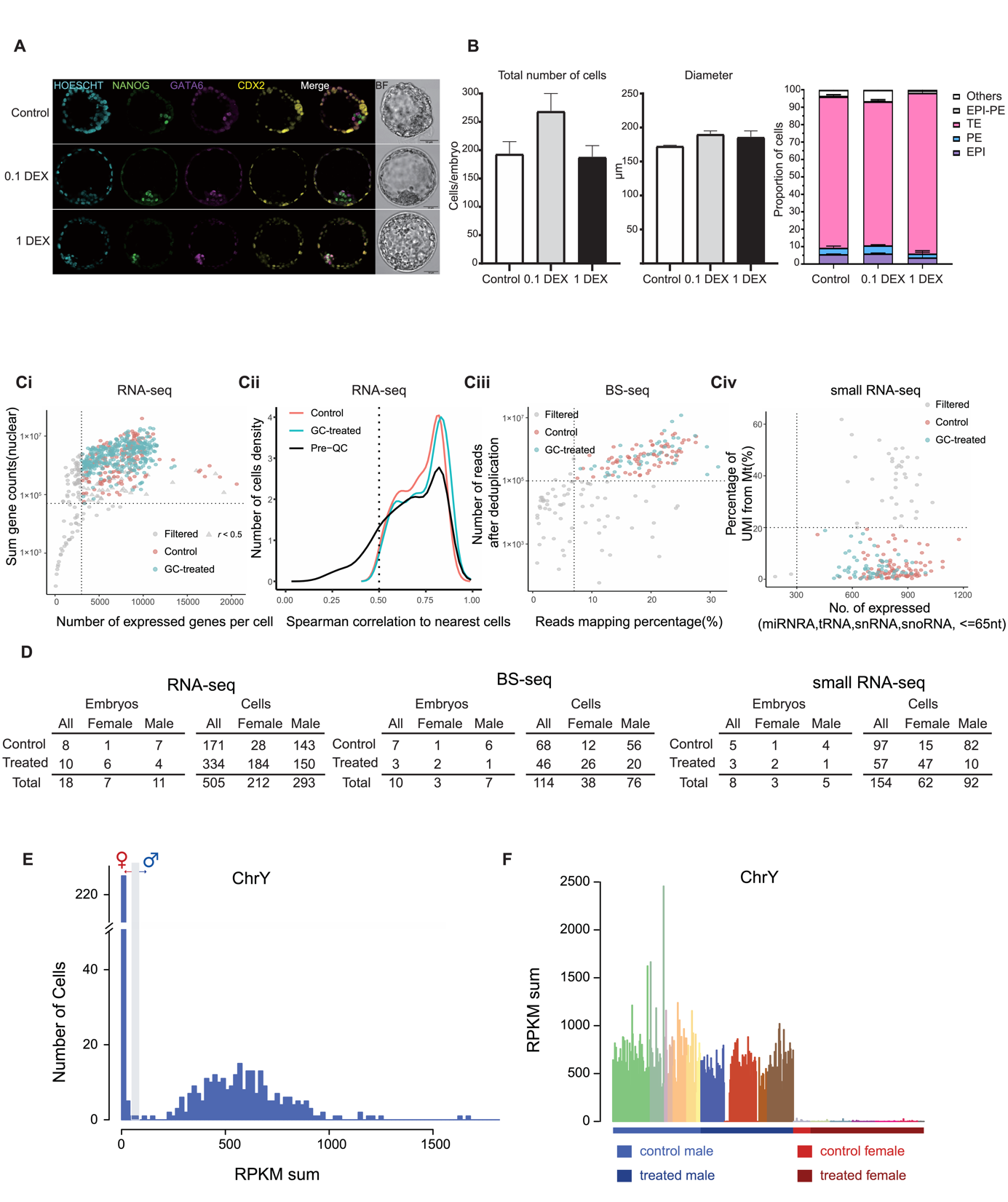
**

**Supplemental Fig. S1.** **Quality control of embryonic cells used in scRNA, scBS and small RNA sequencing analysis.** (A) Representative confocal images of embryos at E7 (control, dexamethasone; (DEX) 0.1 µg/ml and 1 µg/ml) with nuclear marker (Hoescht), and lineage markers for epiblast (NANOG), primitive endoderm (GATA6) and trophectoderm (CDX2). (B) Quantification of the total number of cells, blastocyst size and number of cells within each lineage following treatment and control. (Ci) The number of expressed genes (x-axis) and library size (y-axis) calculated by the sum counts of nuclear genes. Each dot in (C) represents individual cells. Triangles in (C) represent the cells that are further filtered by correlation. The pink and blue dots represent the control and glucocorticoid-treated cells which passed the quality control, respectively. The gray dot denotes the cells that did not pass quality control. The dotted line represents the cutoff used in this analysis. Cells from embryo C6, D9 and D11 have already been removed. (Cii) Distribution of maximum-spearman correlation, using all cell-pairs and all expressed genes. The pink and blue lines represent the control and glucocorticoid-treated cells which passed the scRNA-seq quality control, respectively. The black line denotes the correlation distribution before quality control. The gray dotted line represents the cutoff used in this analysis. (Ciii) Percentage of scBS-seq reads mapping ratio (x-axis) and library size after deduplication (y-axis). (Civ) The number of expressed small RNAs (x-axis) and proportion of UMI from mitochondria (y-axis). (D) The number of cells and embryos for each dataset after quality control. (E) Histogram showing Y Chromosome RPKM sum per cell on the x-axis and cell frequency on the y-axis. Cells with a Y Chromosome RPKM sum less than 50 would be assigned as female, and above 100 as male (see Methods). (F) Barplot of Y Chromosome RPKM sum per cell. Bars are colored by embryo origin.


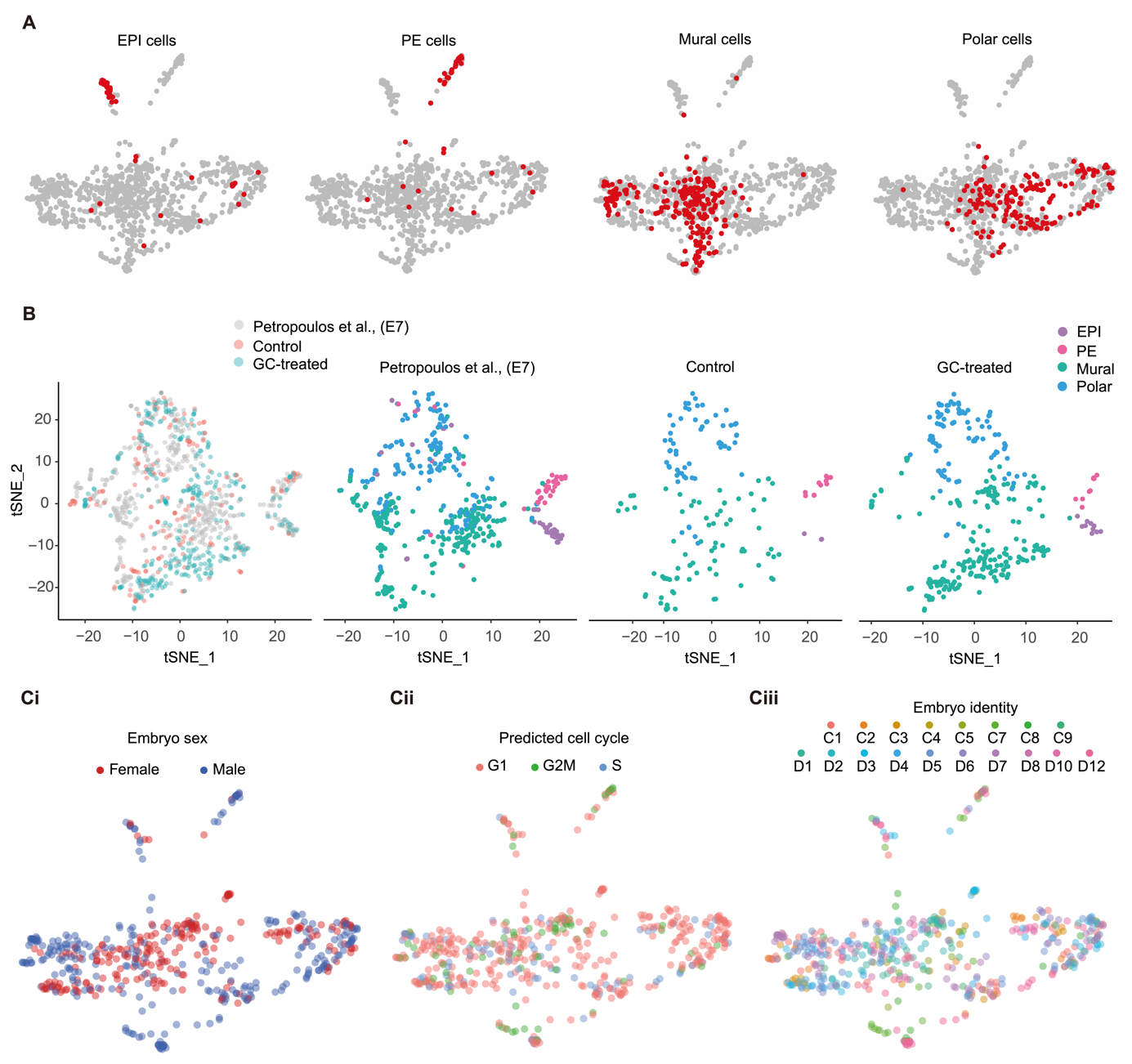


**Supplemental Fig. S2.** **Distribution of cells in the UMAP plot of the integrated dataset.** (A) UMAP embedding of scRNA-seq data with previously annotated E7 cells from Petropoulos et al., 2016 stratified by lineages. Petropoulos et al., 2016 cells are highlighted in red. (B) t-Distributed Stochastic Neighbor Embedding (t-SNE) of the annotated E7 cells from Petropoulos et al., 2016 and this current scRNA-seq dataset. Each dot represents a cell. Cells are colored by treatment and lineage. (C) UMAP embedding of current scRNA-seq dataset. Cells are colored by (Ci) embryo sex, (Cii) predicted cell cycle phase and (Ciii) embryo identity


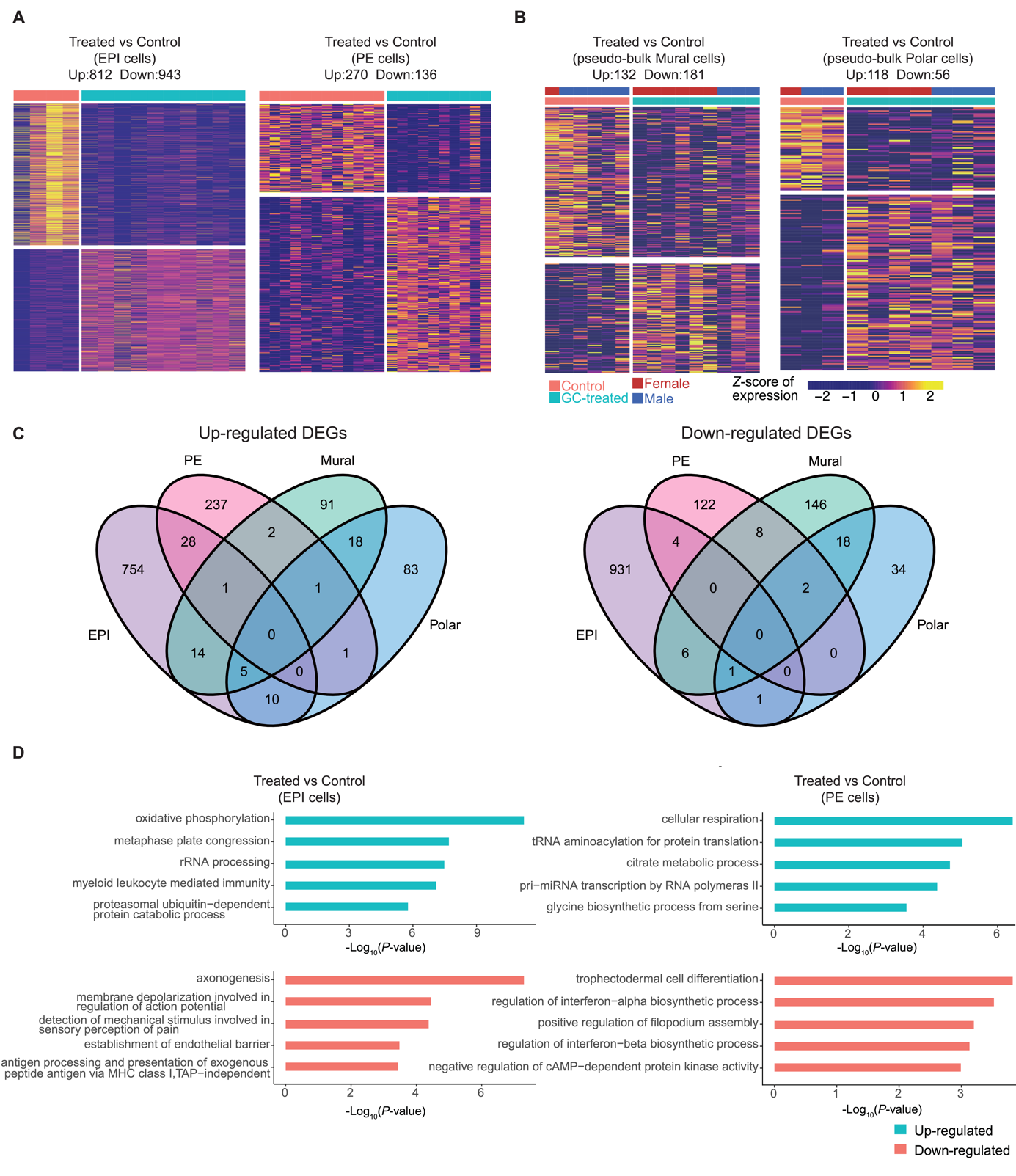


**Supplemental Fig. S3. Differentially expressed genes (DEGs) between control and glucocorticoid-exposed embryos.** (A) Heatmap depicting DEGs between control and glucocorticoid-treated cells for epiblast and primitive endoderm. The number of up and down-regulated DEGs are shown at the top of each plot. (B) Heatmap depicting DEGs between control and glucocorticoid-treated TE cells using pseudo-bulk data by collapsing gene expression per embryo. The number of up and down-regulated DEGs are shown at the top of each plot. (C) Venn diagrams of overlapping DEGs identified in the four lineages. (D) GO enrichment analysis for DEGs caused by glucocorticoid exposure. Top 5 most significantly enriched GO items in the biological process category were shown. Length of the bar represents the significance of overrepresented GO terms. A complete list of GO terms can be viewed in Supplemental Table S3.


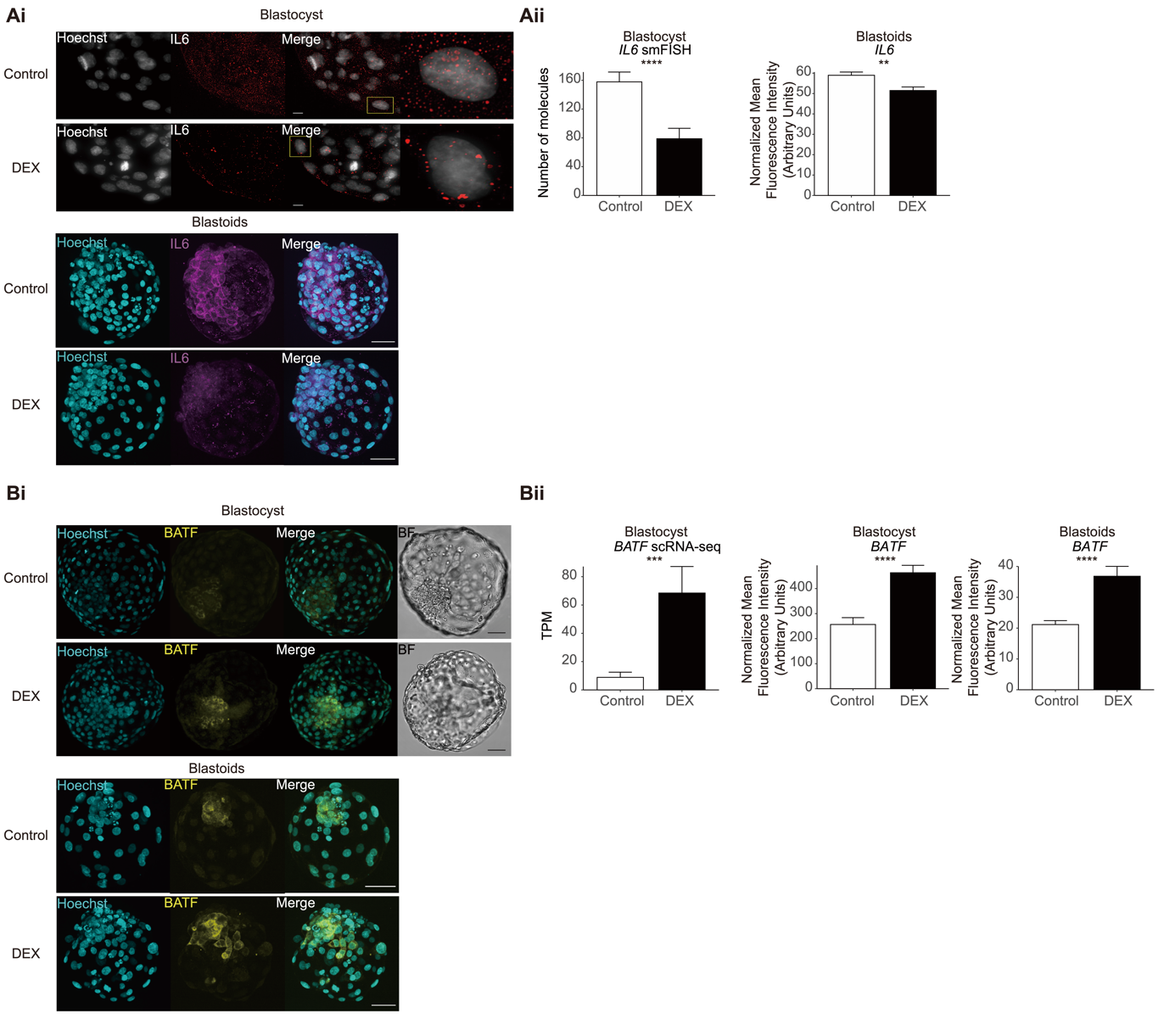


**Supplemental Fig. S4. Validation of selected key differentially expressed genes (DEGs) between control and glucocorticoid-treated embryos and blastoids.** (Ai) Representative smFISH image for *IL6* in the E7 human embryo following control and DEX treatment. Zoomed-in regions show a single nucleus and its associated *IL6* mRNA molecules. Scale bars in all images are set at 10 µm. Below representative z-projections of immunofluorescence staining of blastoids for IL6 following control and DEX treatment. Scale bars in all images are set at 50 µm. (Aii) Quantification of the number of *IL6* mRNA molecules detected in mural cells of the embryo and TPM values obtained from scRNA-seq and IL6 from blastoids. *****P*<0.0001 and ***P*<0.001, Student’s *t*-test, respectively (Bi) Representative z-projections of immunofluorescence staining of human embryos on E7 and human blastoids for BATF following control and DEX treatment. BF=bright field. Scale bars in all images are set at 50 µm. (Bii) Quantification of Normalized Mean Fluorescence Intensity and TPM values obtained from scRNA-seq. ****P<0.0001, Student’s *t*-test and ***FDR < 0.001 calculated using “MAST” test, for immunofluorescence and scRNA-seq, respectively.

**
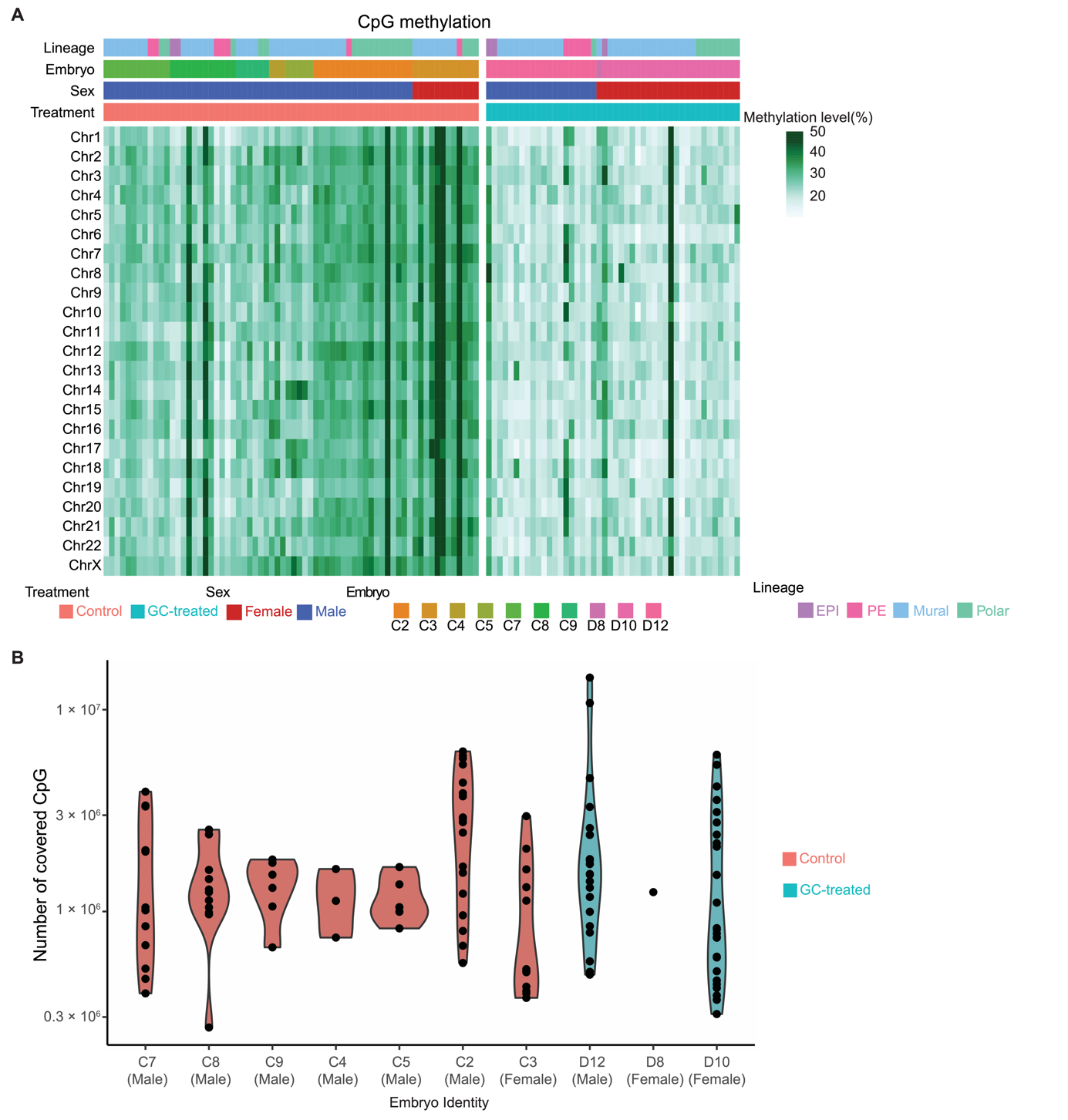
**

**Supplemental Fig. S5. Global methylation and number of sequenced CpGs for scBS-seq reads.** (A) Global methylation level on each chromosome per cell. Cells are ordered by treatment, sex, embryo identity and lineages. (B) Violin plot indicates the number of sequenced CpGs grouped by each embryo. Colors represent the treatment and control.


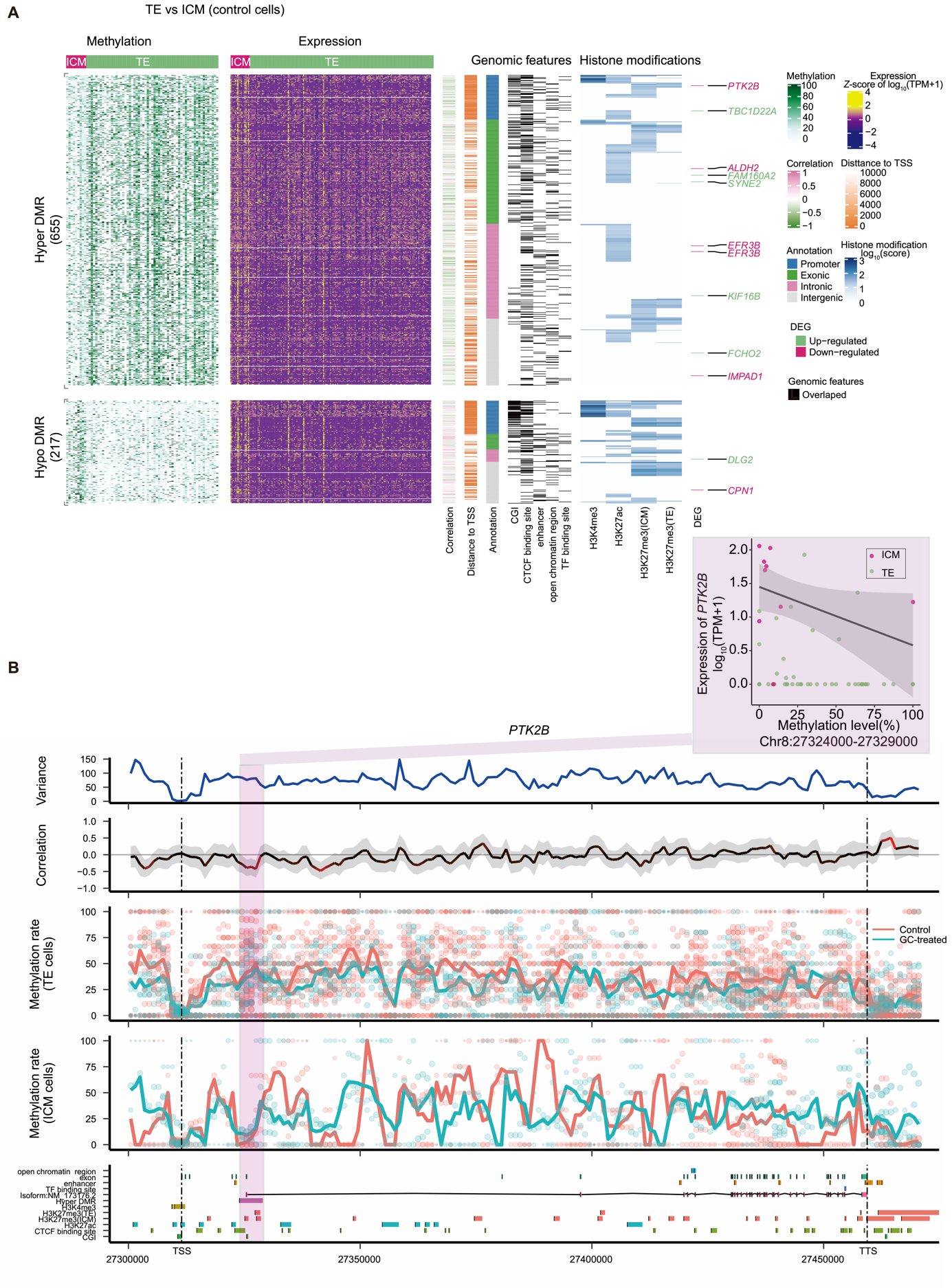


**Supplemental Fig. S6. Differentially methylated regions and genome-wide association between methylation and transcriptional heterogeneity in control of inner cell mass (ICM) and trophectoderm cells (TE).** (A) Comprehensive visualization of associations between methylation, expression, genomic features, and histone modification. From left to right, heatmap represents the methylation level of DMRs in individual cells with the number of DMRs indicated on the left. Missing values in DNA methylation heatmap are indicated with white. The expression level of nearest genes (from transcription start sites (TSS), including different isoforms) for each DMR, the estimated weighted correlation between the DMRs’ methylation level and gene expression, the distance to its nearest TSS, the DMR annotation, the corresponding overlapping genomic features, the histone modification scores for the DMR regions. Genes highlighted are the DEGs associated with the DMRs, where pink represents down-regulated expression and green represents up-regulated gene expression. (B) Representative methylation variance, correlation, and methylation rate near the 3’ region of *PTK2B*. Shown from bottom to top are the annotation of the PTK2B locus with genomic features, histone modifications; the estimated methylation level of 3 kb sliding windows for each cell with dot size indicating CpG coverage, dot colors indicating different treatments. The solid curve denoting the weighted mean methylation rate with line colors representing different treatments and dashed vertical lines delineating the position of transcription termination sites (TTS) of *PTK2B*. The correlation between the methylation rate and *PTK2B* expression for each window. Color of the curve represents the level of significance for the correlations and the gray shaded area denotes the 95% confidence interval of the correlation coefficient using the estimated weighted DNA-methylation variance between cells. The scatter plot at the top right depicts the correlation between DNA methylation at the selected DMR (outlined in purple) and *PTK2B* expression.
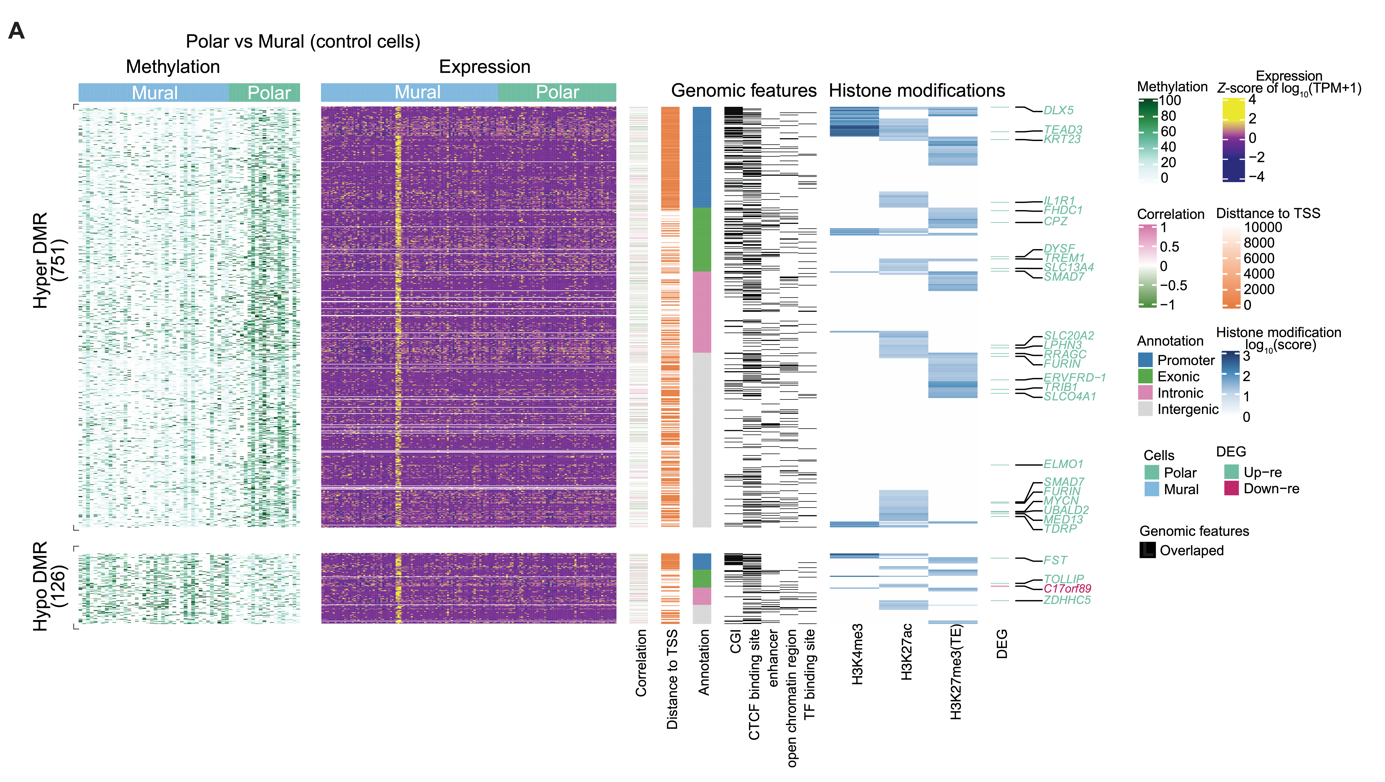


**Supplemental Fig. S7.** **Differentially methylated regions and genome-wide association between methylation and transcriptional heterogeneity amongst trophectoderm (TE) cells**. Comprehensive correspondence between methylation, expression, genomic features, and histone modification. Cells depicted and DMRs identified are from the mural and polar cells in control embryos. From left to right, heatmap represents the methylation level of DMRs in individual cells with the number of DMRs indicated on the left. Missing values in DNA methylation heatmap are indicated with white. The expression level of nearest genes (from TSS, including different isoforms) for each DMR, the estimated weighted correlation between the DMRs’ methylation level and gene expression, the distance to its nearest TSS, the DMR annotation, the corresponding overlapping genomic features, the histone modification scores for the DMR regions. Genes highlighted are the DEGs associated with the DMRs, where red represents up-regulated expression in mural cells and green represents up-regulated gene expression in polar cells.

**
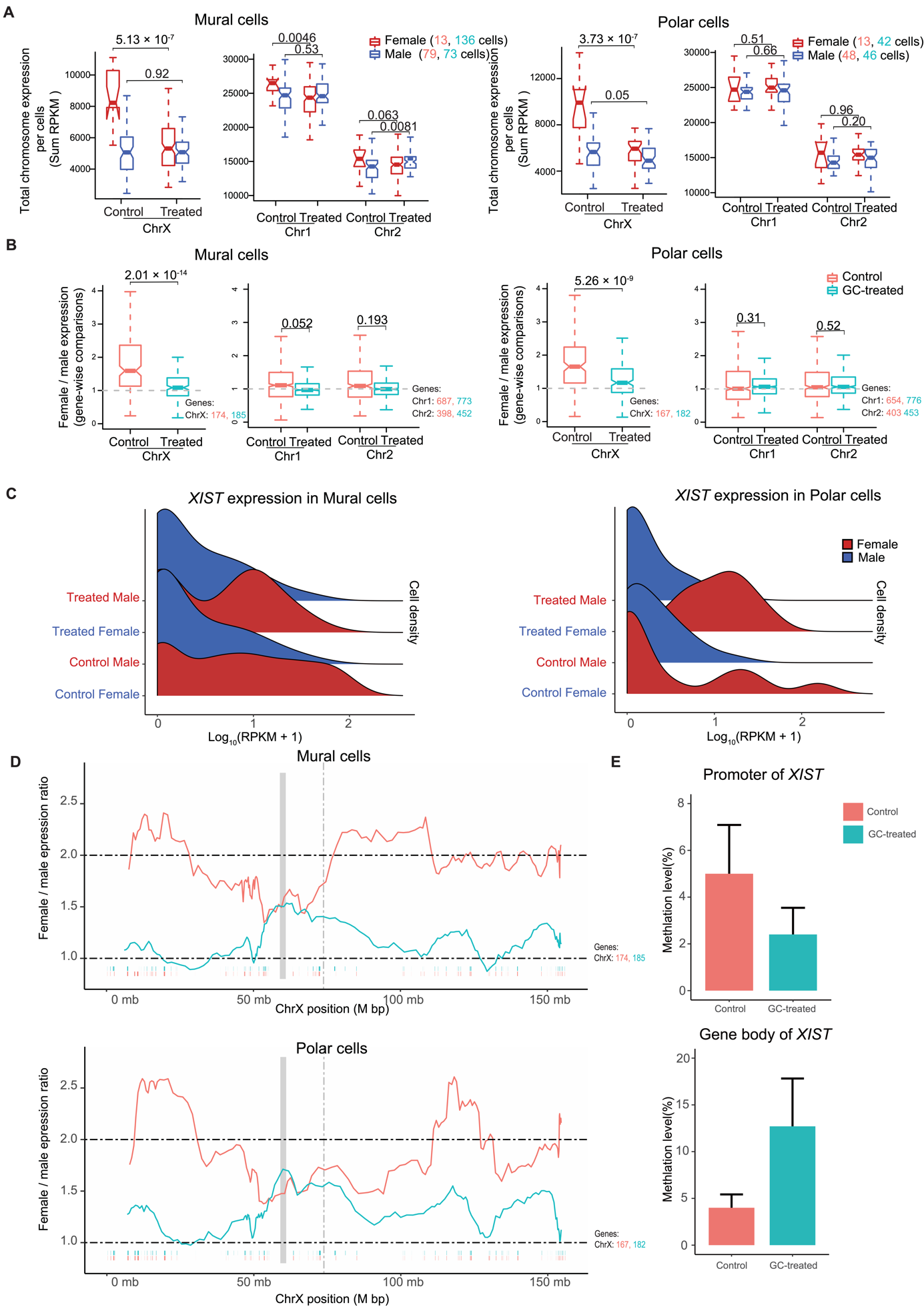
**

**Supplemental Fig. S8. Impact of glucocorticoid exposure on X Chromosome activity in mural and polar cells and DNA methylation of *XIST*.** (A) Boxplots of X Chromosome and Chromosome 1 and 2 RPKM sums stratified by sex, treatment, and lineages. Color represents sex. (B) Boxplots of female-to-male expression ratios of X Chromosome and Chromosome 1 and 2 linked genes in mural and polar cells. Color represents treatment and control. All *P*-values were calculated using the two-sided Wilcoxon test. (C) Ridge plot for *XIST* expression stratified by sex and treatment in mural and polar cells. Color represents sex. (D) Sliding window (20-nearest-genes) of female-to-male expression average along the X Chromosome for mural and polar cells. The ticks below the moving-average lines indicate the location of expressed genes included in the estimates, colored according to different treatments. The gray block represents the locus of the centromere position. The gray dashed line denotes the locus of *XIST*. (E) Weighted mean methylation level at promoter and gene body region of *XIST* in female TE cells. Error bar represents standard error of the mean (SEM).


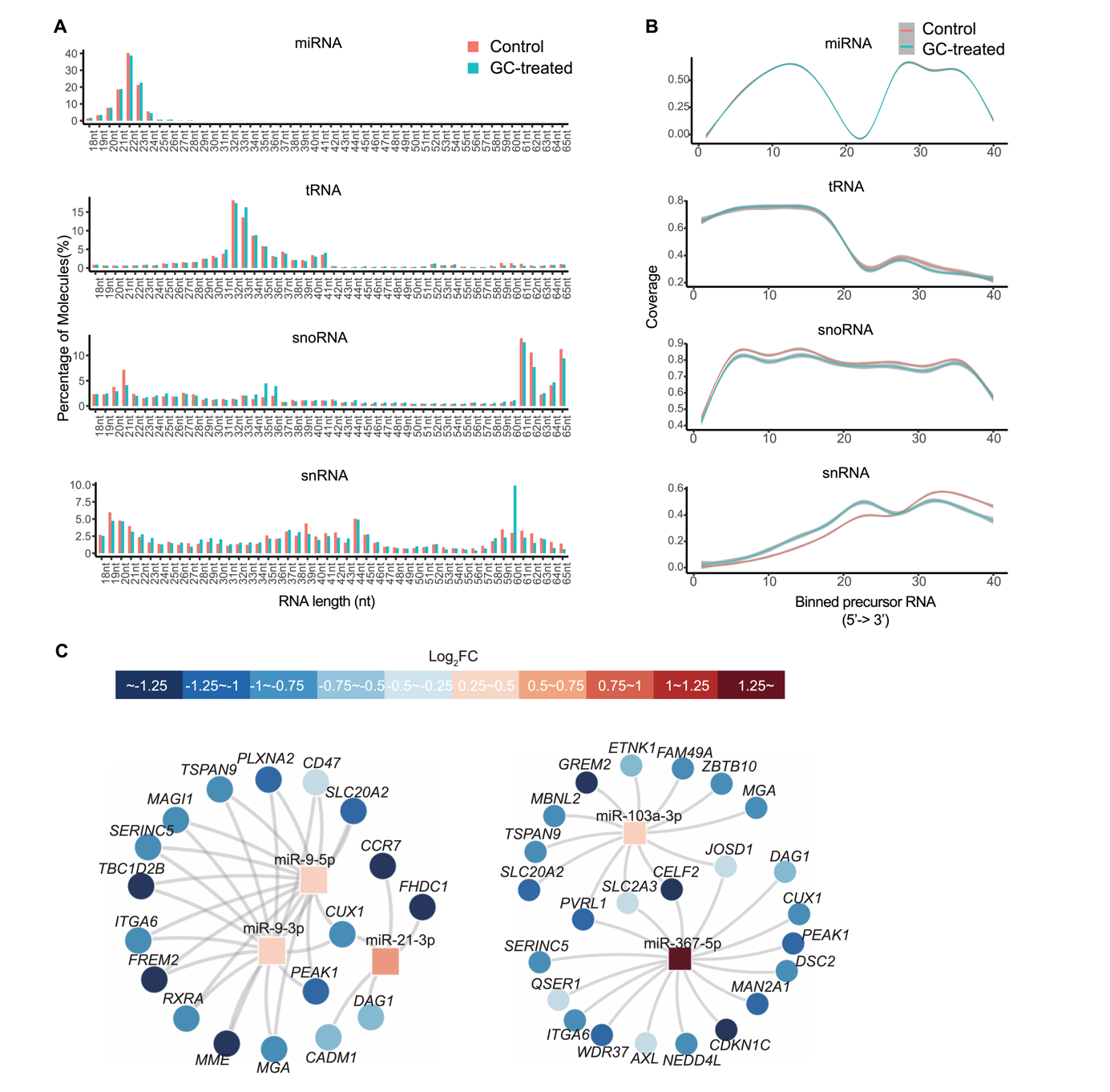


**Supplemental Fig. S9.** **Characterization of single-cell small RNA sequencing and additional miRNA-mRNA co-expression regulatory networks identified.** (A) Size distributions of small RNAs captured in single human embryo cells. (B) Coverage of small RNAs across the length of the precursor RNAs (divided into 40 bins). The gray shaded area denotes the 95% confidence interval of the coverage. (C) Additional node-link diagrams for differentially expressed miRNA-mRNA co-expression regulatory networks. Red and blue represent log2 fold changes of expression between control and treated cells.

**
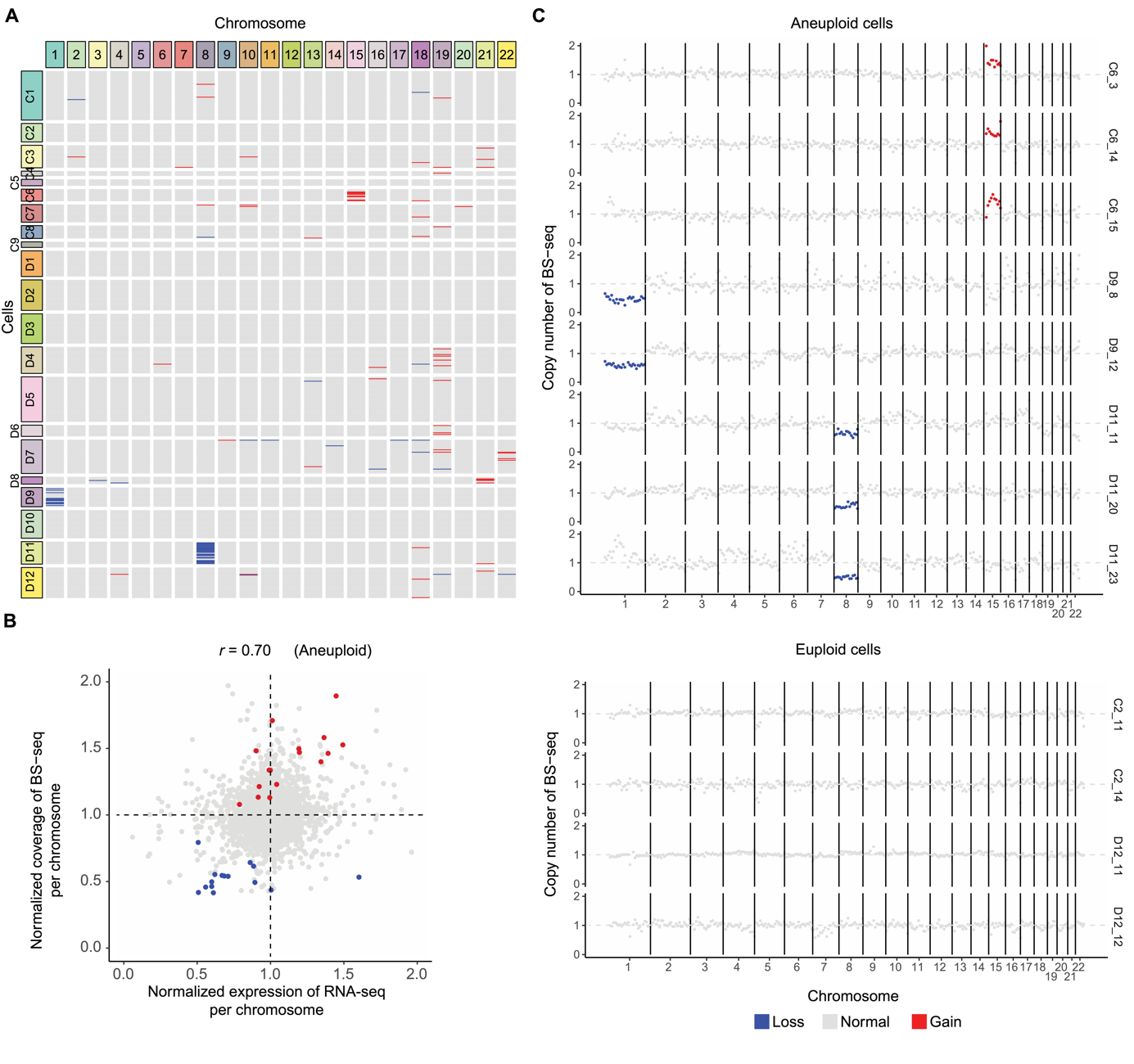
**

**Supplemental Fig. S10.** **Aneuploidy inference on scRNA-seq data and in parallel, methylation data.** (A) Copy number variation (CNV) for each individual cell of different embryos.  Each row represents a cell and each column represents chromosomes. The CNV status is indicated by the color of lines. (B) Correlation between normalized expression and normalized reads coverage of each chromosome. Normalized expression of each chromosome was calculated by the sum of transcripts per million (TPM) divided by the TPM sum for that chromosome across all samples. Likewise, normalized read coverage was calculated from scBS-seq data coverage per chromosome. Spearman correlation was calculated by aneuploidy chromosome expression and corresponding reads coverage. (C) Representative examples of scBS-seq data coverage in aneuploidy and euploidy single-cell samples. Abnormal copy numbers are highlighted in duplication or deletion inferred from scRNA-seq data. (A, B, C) Red represents duplication, blue represents deletion and gray represents diploid.

**Reference**

Alexa A, Rahnenfuhrer J. 2016. topGO: Enrichment analysis for Gene Ontology. R package version 2.28. 0. *BioConductor Publ online*.

Anders S, Huber W. Differential expression of RNA-Seq data at the gene level-the DESeq package.

Angermueller C, Clark SJ, Lee HJ, Macaulay IC, Teng MJ, Hu TX, Krueger F, Smallwood SA, Ponting CP, Voet T, et al. 2016. Parallel single-cell sequencing links transcriptional and epigenetic heterogeneity. *Nat Methods* **13**: 229–232.

Chan PP, Lowe TM. 2016. GtRNAdb 2.0: an expanded database of transfer RNA genes identified in complete and draft genomes. *Nucleic Acids Res* **44**: D184-9.

Clark SJ, Argelaguet R, Kapourani CA, Stubbs TM, Lee HJ, Alda-Catalinas C, Krueger F, Sanguinetti G, Kelsey G, Marioni JC, et al. 2018. ScNMT-seq enables joint profiling of chromatin accessibility DNA methylation and transcription in single cells e. *Nat Commun* **9**: 1–9.

Ewels PA, Peltzer A, Fillinger S, Patel H, Alneberg J, Wilm A, Garcia MU, Di Tommaso P, Nahnsen S. 2020. The nf-core framework for community-curated bioinformatics pipelines. *Nat Biotechnol* **38**: 276–278.

Faridani OR, Abdullayev I, Hagemann-Jensen M, Schell JP, Lanner F, Sandberg R. 2016. Single-cell sequencing of the small-RNA transcriptome. *Nat Biotechnol* **34**: 1264–1266.

Finak G, McDavid A, Yajima M, Deng J, Gersuk V, Shalek AK, Slichter CK, Miller HW, McElrath MJ, Prlic M, et al. 2015. MAST: a flexible statistical framework for assessing transcriptional changes and characterizing heterogeneity in single-cell RNA sequencing data. *Genome Biol* **16**: 278.

González R, Ruiz-León Y, Gomendio M, Roldan ERS. 2010. The effect of glucocorticoids on mouse oocyte in vitro maturation and subsequent fertilization and embryo development. *Toxicol In Vitro* **24**: 108–115.

Gu Z, Eils R, Schlesner M. 2016. Complex heatmaps reveal patterns and correlations in multidimensional genomic data. *Bioinformatics* **32**: 2847–2849.

Guo G, von Meyenn F, Rostovskaya M, Clarke J, Dietmann S, Baker D, Sahakyan A, Myers S, Bertone P, Reik W, et al. 2017. Epigenetic resetting of human pluripotency. *Development* **144**: 2748–2763.

Haeussler M, Zweig AS, Tyner C, Speir ML, Rosenbloom KR, Raney BJ, Lee CM, Lee BT, Hinrichs AS, Gonzalez JN, et al. 2019. The UCSC Genome Browser database: 2019 update. *Nucleic Acids Res* **47**: D853–D858.

Hafemeister C, Satija R. 2019. Normalization and variance stabilization of single-cell RNA-seq data using regularized negative binomial regression. *Genome Biol* **20**: 296.

Hagemann-Jensen M, Abdullayev I, Sandberg R, Faridani OR. 2018. Small-seq for single-cell small-RNA sequencing. *Nat Protoc* **13**: 2407–2424.

Harrow J, Frankish A, Gonzalez JM, Tapanari E, Diekhans M, Kokocinski F, Aken BL, Barrell D, Zadissa A, Searle S, et al. 2012. GENCODE: the reference human genome annotation for The ENCODE Project. *Genome Res* **22**: 1760–1774.

Kagawa H, Javali A, Khoei HH, Sommer TM, Sestini G, Novatchkova M, Scholte Op Reimer Y, Castel G, Bruneau A, Maenhoudt N, et al. 2022. Human blastoids model blastocyst development and implantation. *Nature* **601**: 600–605.

Kozomara A, Birgaoanu M, Griffiths-Jones S. 2019. miRBase: from microRNA sequences to function. *Nucleic Acids Res* **47**: D155–D162.

Krueger F. 2015. Trim galore. *A wrapper tool around Cutadapt FastQC to consistently apply Qual Adapt trimming to FastQ files* **516**: 517.

Krueger F, Andrews SR. 2011. Bismark: a flexible aligner and methylation caller for Bisulfite-Seq applications. *Bioinformatics* **27**: 1571–1572.

Kuleshov M V, Jones MR, Rouillard AD, Fernandez NF, Duan Q, Wang Z, Koplev S, Jenkins SL, Jagodnik KM, Lachmann A, et al. 2016. Enrichr: a comprehensive gene set enrichment analysis web server 2016 update. *Nucleic Acids Res* **44**: W90-7.

Martin M. 2011. Cutadapt removes adapter sequences from high-throughput sequencing reads. *EMBnet J* **17**: 10–12.

Petropoulos S, Edsgard D, Reinius B, Deng Q, Panula SP, Codeluppi S, Plaza Reyes A, Linnarsson S, Sandberg R, Lanner F. 2016. Single-Cell RNA-Seq Reveals Lineage and X Chromosome Dynamics in Human Preimplantation Embryos. *Cell* **165**: 1012–1026.

Rouillard AD, Gundersen GW, Fernandez NF, Wang Z, Monteiro CD, McDermott MG, Ma’ayan A. 2016. The harmonizome: a collection of processed datasets gathered to serve and mine knowledge about genes and proteins. *Database (Oxford)* **2016**.

Santana PPB, Carvalho CMF, da Costa NN, Silva TVG, Ramos PCA, Cordeiro MS, Santos SSD, Khayat AS, Ohashi OM, Miranda MS. 2014. Effect of dexamethasone on development of in vitro-produced bovine embryos. *Theriogenology* **82**: 10–6.

Soneson C, Robinson MD. 2018. Bias, robustness and scalability in single-cell differential expression analysis. *Nat Methods* **15**: 255–261.

Tickle T, TI GC, Brown M, Haas B. 2019. inferCNV of the Trinity CTAT Project. *Klarman Cell Obs Broad Inst MIT Harvard, Cambridge, MA, USA*.

Xia W, Xu J, Yu G, Yao G, Xu K, Ma X, Zhang N, Liu B, Li T, Lin Z, et al. 2019. Resetting histone modifications during human parental-to-zygotic transition. *Science (80- )* **365**: 353–360.

Yu G, Wang LG, Han Y, He QY. 2012. clusterProfiler: an R package for comparing biological themes among gene clusters. *OMICS* **16**: 284–287.

Yu G, Wang LG, He QY. 2015. ChIPseeker: an R/Bioconductor package for ChIP peak annotation, comparison and visualization. *Bioinformatics* **31**: 2382–2383.

Zerbino DR, Wilder SP, Johnson N, Juettemann T, Flicek PR. 2015. The ensembl regulatory build. *Genome Biol* **16**: 56.

Zhou F, Wang R, Yuan P, Ren Y, Mao Y, Li R, Lian Y, Li J, Wen L, Yan L, et al. 2019. Reconstituting the transcriptome and DNA methylome landscapes of human implantation. *Nature* **572**: 660–664.
